# Synthesis, crystal structures, and Hirshfeld analysis of three hexa­hydro­quinoline derivatives

**DOI:** 10.1107/S2056989022009495

**Published:** 2022-10-04

**Authors:** Scott A. Steiger, Chun Li, Allen G. Oliver, Nicholas R. Natale

**Affiliations:** a Department of Biomedical and Pharmaceutical Sciences, University of Montana, Missoula, MT 59812, USA; b Department of Chemistry, Ithaca College, 953 Danby Road, Ithaca, NY 14850, USA; c Department of Chemistry and Biochemistry, University of Notre Dame, Notre Dame, IN 46556, USA; Texas A & M University, USA

**Keywords:** crystal structure, hydrogen bonding, hexa­hydro­quinoline (HHQ), 1,4-di­hydro­pyridine (1,4-DHP), calcium-channel antag­onist, multi-drug resistance (MDR)

## Abstract

Three hexa­hydro­quinoline derivatives were synthesized and crystallized in an effort to study the structure–activity relationships of these calcium-channel antagonists. The derivatives are ethyl 4-(2-meth­oxy­phen­yl)-2,7,7-trimethyl-5-oxo-1,4,5,6,7,8-hexa­hydro­quinoline-3-carboxyl­ate, C_22_H_27_NO_4_, (I), ethyl 4-(4-meth­oxy­phen­yl)-2,7,7-trimethyl-5-oxo-1,4,5,6,7,8-hexa­hydro­quinoline-3-carb­ox­yl­ate, C_22_H_27_NO_4_, (II), and ethyl 4-(3,4-di­hydroxy­phen­yl)-2,7,7-trimethyl-5-oxo-1,4,5,6,7,8-hexa­hydro­quinoline-3-carboxyl­ate, C_21_H_24_NO_5_, (III). In these hexa­hydro­quinoline derivatives, common structural features such as a flat-boat conformation of the 1,4-di­hydro­pyridine (1,4-DHP) ring, an envelope conformation of the fused cyclo­hexa­none ring, and a substituted phenyl group at the pseudo-axial position are retained. Hydrogen bonds are the main contributors to the packing of the mol­ecules in these crystals.

## Abstract 2

Three hexa­hydro­quinoline derivatives were synthesized and crystallized in an effort to study the structure–activity relationships of these calcium-channel antagonists. n these hexa­hydro­quinoline derivatives, common structural features such as a flat-boat conformation of the 1,4-di­hydro­pyridine (1,4-DHP) ring, an envelope conformation of the fused cyclo­hexa­none ring, and a substituted phenyl group at the pseudo-axial position are retained. Hydrogen bonds are the main contributors to the packing of the mol­ecules in these crystals.

## 1. Chemical context

4-Aryl-1,4-di­hydro­pyridines (DHPs) that bind the L-type voltage-gated calcium channels (VGCC) have been applied in general medical practice for over three decades (Zamponi, 2016 ▸). Many modifications on 1,4-DHP have been performed to obtain active compounds such as calcium-channel agonists or antagonists (Martín *et al.*, 1995 ▸; Rose, 1990 ▸; Rose & Dräger, 1992 ▸; Trippier *et al.*, 2013 ▸). One such modification is fusing a cyclo­hexa­none ring to form hexa­hydro­quinoline (HHQ), in which the orientation of the carbonyl group of the ester substituent at the 5-position in the 1,4-DHP ring has been fixed. This class of compounds has been shown to have calcium-channel antagonistic activity (Aygün Cevher *et al.*, 2019 ▸), inhibit the multidrug-resistance transporter (MDR) (Shahraki *et al.*, 2017 ▸, 2020 ▸), as well as possessing anti-inflammatory and stem-cell differentiation properties, and have been implicated in slowing neurodegenerative disorders (Trippier *et al.*, 2013 ▸). Recently, specific substitutions of the cyclo­hexenone ring were found to have distinct selectivity profiles to different calcium channel subtypes (Schaller *et al.*, 2018 ▸). Another report also showed that the 4-aryl-hexa­hydro­quinolines, especially the ones containing a meth­oxy moiety, exhibit good anti­oxidant property as radical scavengers (Yang *et al.*, 2011 ▸). In a continuation of our study on the structure–activity relationship of this class of 4-aryl-hexa­hydro­quinolines (Steiger *et al.*, 2014 ▸, 2018 ▸, 2020 ▸), and to understand stereoelectronic effects, which define selectivity, as well as to explore the scope and limitations of our synthetic methodologies (Steiger *et al.*, 2016 ▸), we report herein the crystal structures of three 4-aryl-hexa­hydro­quinoline derivatives. 
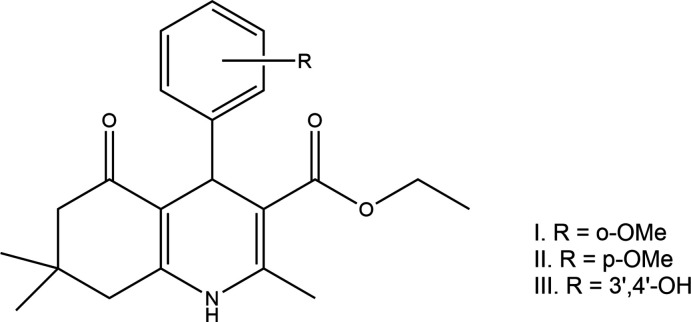


## 2. Structural commentary

**Figure 1:**
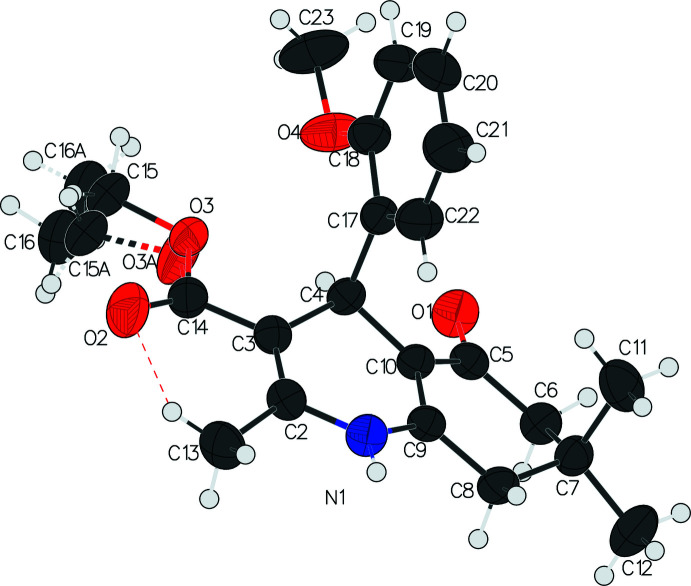
The asymmetric unit of compound I showing the atom-labeling scheme. Displacement ellipsoids are drawn at the 50% probability level. The intra­molecular hydrogen bond between C13—H13*B* and O2 is shown as a dashed line. The crystal disintegrated below 273 K and the X-ray structure was acquired at room temperature.

**Figure 2:**
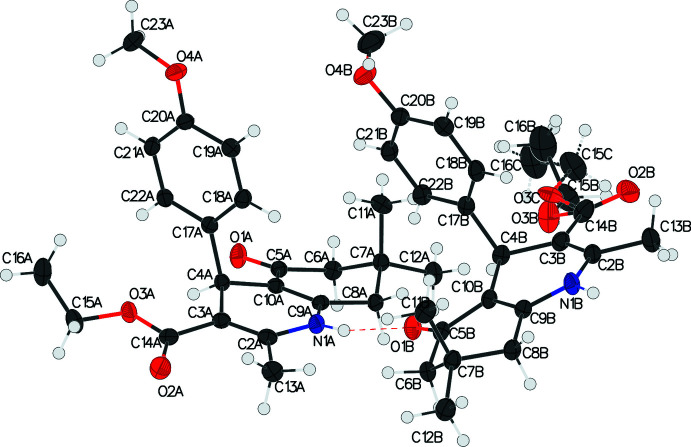
The asymmetric unit of compound II showing the atom-labeling scheme. Displacement ellipsoids are drawn at the 50% probability level. The inter­molecular hydrogen bond between N1*A*—H1*A* and O1*B* is shown as a dashed line.

**Figure 3:**
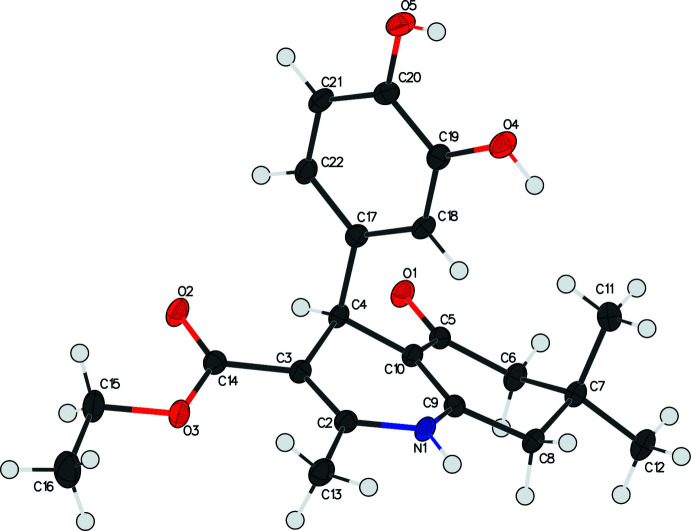
The asymmetric unit of compound III showing the atom-labeling scheme. Displacement ellipsoids are drawn at the 50% probability level.

The asymmetric unit of the title compound I contains one independent mol­ecule, which crystallizes in the triclinic *P* 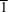 space group (Fig. 1 ▸). Compounds II and III both crystallize in the monoclinic space group *P*2_1_/*n*. The asymmetric unit of compound II contains two independent mol­ecules, *A* and *B* (Fig. 2 ▸), while compound III has only one independent mol­ecule in the asymmetric unit (Fig. 3 ▸). Similar to the other 4-aryl-hexa­hydro­quinoline derivatives that we have reported (Steiger, *et al.*, 2014 ▸; 2018 ▸; 2020 ▸), compounds I, II, and III all share the common structural features such as a flattened boat conformation on the 1,4-DHP ring, envelope conformation of the cyclo­hexa­none ring, and the pseudo-axial position of the 4-aryl group.

The shallow-boat confirmation of the 1,4-DHP ring is one of the factors that leads to higher calcium-channel activity (Linden *et al.*, 2004 ▸) The shallowness of the boat conformation in these three compounds are indicated by the marginal displacements of atom N1 and C4 from the mean plane (the base of the boat) defined by the two double bonds (C2=C3 and C9=C10). The distances between N1 and the mean plane formed by C2/C3/C9/C10 are 0.159 (3), 0.110 (2), 0.110 (3), and 0.181 (2) Å for compounds I, IIA, IIB, and III, respectively. The corresponding distances between C4 and the same mean plane are 0.341 (3), 0.295 (3), 0.253 (3), and 0.399 (2) Å for compounds I, IIA, IIB, and III, respectively.

The pseudo-axial position of the C4-aryl group to the 1,4-DHP ring is another key factor that is essential for pharmacological activity (Langs *et al.*, 1987 ▸). In the title compounds, the substituted phenyl rings are almost orthogonal to the base of the 1,4-DHP ring, with the mean plane normal to normal angles being 89.09 (7), 92.52 (6), 93.52 (6), and 90.59 (5)° for compounds I, IIA, IIB, and III, respectively (see Table 1 ▸ for calculated parameters). It is noteworthy that the *para*-meth­oxy group on the phenyl ring is flexible and can be either *anti-* or *syn-* periplanar to the H atom on C4, *i.e.* pointing either to (IIA) or away from (IIB) the 1,4-DHP ring.

In all three compounds, the cyclo­hexa­none rings adopt the envelope conformation, which can be qu­anti­fied using Cremer & Pople’s ring-puckering parameters. Ideally, the envelope conformation would have θ = 54.7° (or θ =125.3° in the case of an absolute configuration change) and φ = *n* × 60°. The θ and φ values of the title compounds are very close to the ideal angles with deviations less than 10° and are listed in Table 2 ▸.

Although the carbonyl on the ester group is conjugated to the adjacent endocyclic double bond and is co-planar to the 1,4-DHP mean plane, the whole ester group is flexible. The C=O bond can be either *cis* (I, IIA and IIB) or *trans* (III) to the adjacent double bond, and the extended or curled orientations of the ethyl group are observed in these crystal structures. The disordered ethyl groups in compound I and compound II also indicate the flexibility of the ester group.

## 3. Supra­molecular features

**Figure 4:**
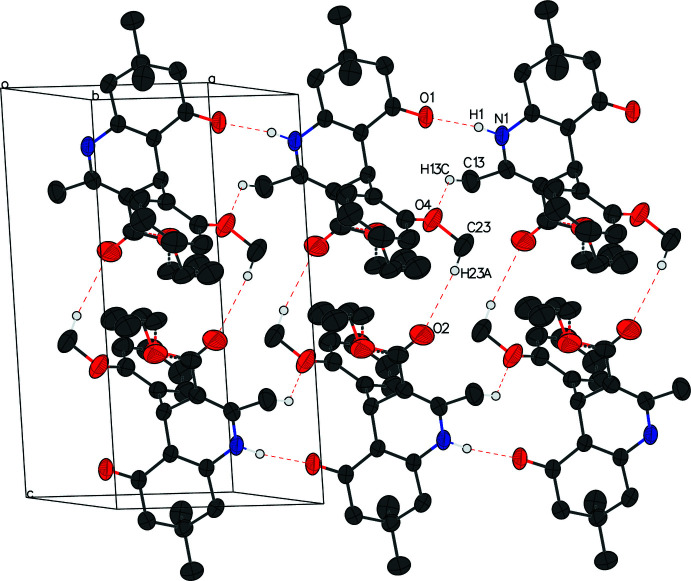
The packing of compound I. Inter­molecular hydrogen bonds are shown as dashed lines, and H atoms not involved in these hydrogen bonds are removed for clarity.

In compound I, hydrogen bonds between N1—H1 and O1 form a chain perpendicular to the (100) plane. Short contact C23—H23*A*⋯O2 links alternate enanti­omers to form a pair perpendicular to the (001) plane (Table 3 ▸, Fig. 4 ▸).

**Figure 5:**
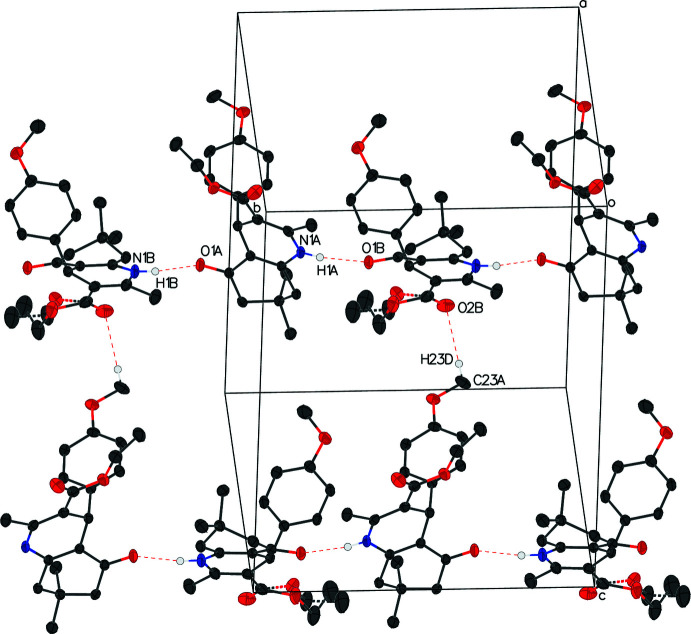
The packing of title compound **II**. H atoms bonds are shown as dashed lines. H atoms not involved in these hydrogen bonds are removed for clarity.

In compound II, hydrogen bonds N1*A*—H1*A*⋯O1*B* and N1*B*—H1*B*⋯O1*A* link the two independent mol­ecules *A* and *B* to form a chain perpendicular to the (010) plane. Close contacts C23*B*—H23*B*⋯O2*A* and C23*A*—H23*D*⋯O2*B* link the two independent mol­ecules zigzaggedly along the *c-*axis direction (Table 4 ▸, Fig. 5 ▸).

**Figure 6:**
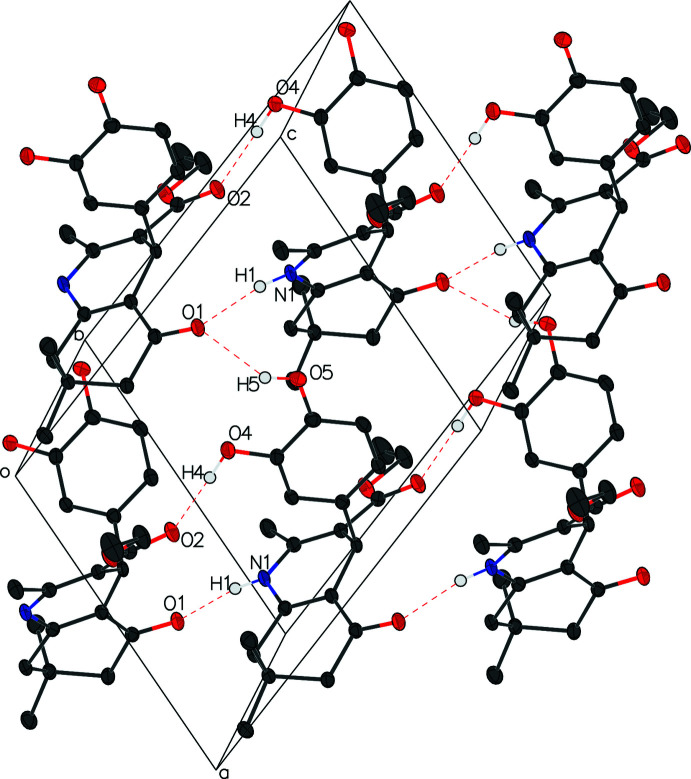
The packing of title compound **III**. Inter­molecular hydrogen bonds (shown in dashed lines) cross link the mol­ecules to form a sheet parallel to the (010) plane. H atoms not involved in these hydrogen bonds are removed for clarity.

In compound III, a chain is formed by hydrogen bonds N1—H1⋯O1^i^ and O4—H4⋯O2^i^ between alternating enanti­omers and runs perpendicular to the (101) plane. Hydrogen bond O5—H5⋯O1^ii^ links the mol­ecules in a chain perpendicular to the (100) plane and cross-links the other chain to form a sheet of mol­ecules parallel to the (010) plane (Table 5 ▸, Fig. 6 ▸).

## 4. Hirshfeld surface analysis

**Figure 7:**
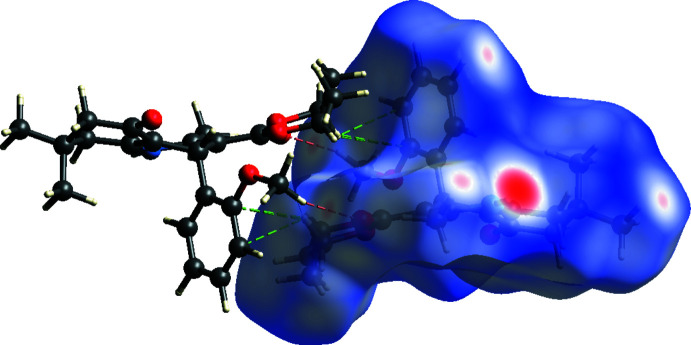
Hirshfeld surface of **I** mapped over *d*_norm_. Short and long contacts are indicated as red and blue regions, respectively. Contacts with distances approximately equal to the sum of the van der Waals radii are colored white. A σ- π inter­action between C15—H15 and phenyl ring is shown as green dashed lines. Hydrogen bond C23—H23*A*⋯O2 is shown as red dashed lines.

**Figure 8:**
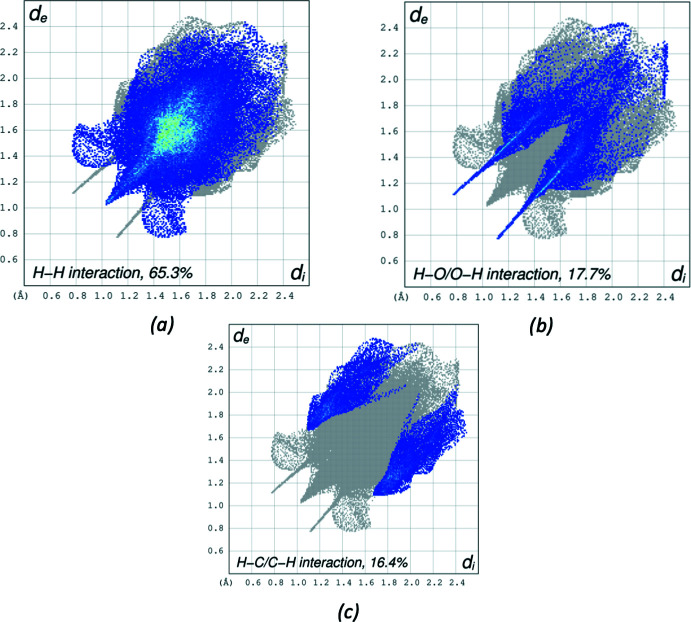
The two-dimensional fingerprint plots for I delineated into (*a*) H⋯H contacts, (*b*) H⋯O/O⋯H contacts, (*c*) H⋯C/C⋯H contacts. Other contact contributions less than 1% are omitted.

Hirshfeld surface analysis (Spackman & Jayatilaka, 2009 ▸) was performed, and the associated two-dimensional fingerprint plots (McKinnon *et al.*, 2007 ▸) were generated to qu­antify the inter­molecular inter­actions using *Crystal Explorer 21.5* (Spackman *et al.*, 2021 ▸). The Hirshfeld surface of the title compound I is mapped over *d*_norm_ in a fixed color scale of −0.5596 (red) to 1.4022 (blue) arbitrary units (Fig. 7 ▸). The N—H⋯O hydrogen bond is apparent as red spots on the surface. A σ–π inter­action between the ester ethyl group and the phenyl ring is noticeable. The delineated two-dimensional fingerprint plots (Fig. 8 ▸) show that the contributions to the overall Hirshfeld surface area arise from H⋯H contacts (65.3%), O⋯H/H⋯O contacts (17.7%), and C⋯H/H⋯C inter­actions (16.4%).

**Figure 9:**
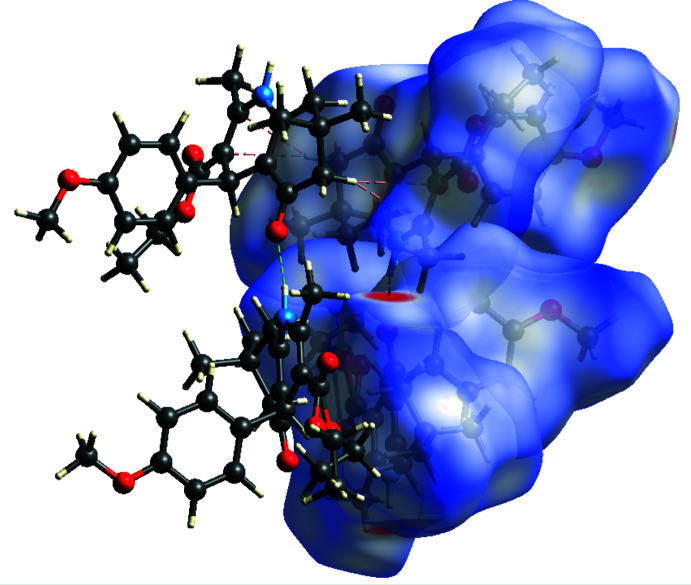
Hirshfeld surface of II mapped over *d*_norm_. Short and long contacts are indicated as red and blue regions, respectively. Contacts with distances approximately equal to the sum of the van der Waals radii are colored white. A σ–π inter­action (C6*A*—H6*AB* to double bond C2*A*=C3*A*) is shown as red dashed lines. Hydrogen bonds between N—H and O are shown as green dashed lines.

**Figure 10:**
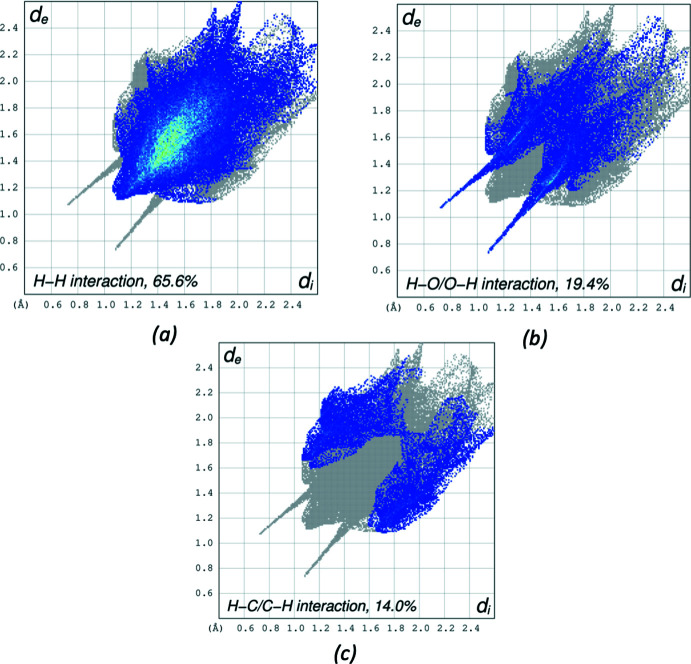
The two-dimensional fingerprint plots for II delineated into (*a*) H⋯H contacts, (*b*) H⋯O/O⋯H contacts, (*c*) H⋯C/C⋯H contacts. Other contact contributions of less than 1% are omitted.

For compound II, the Hirshfeld surface analysis was performed with two independent mol­ecules, in a fixed color scale of −0.6119 (red) to 1.7055 (blue) arbitrary units. In addition to hydrogen bonds, σ–π inter­actions are also identifiable between C6*A*—H6*AB* and double bond C2*A*—C3*A* (Fig. 9 ▸). The delineated two-dimensional fingerprint plots shown in Fig. 10 ▸ indicate that H⋯H contacts (65.6%) make the main contribution to the overall Hirshfeld surface area. The O⋯H/H⋯O contacts and C⋯H/H⋯C inter­actions contribute 19.4% and 14.0% of the Hirshfeld surface, respectively.

**Figure 11:**
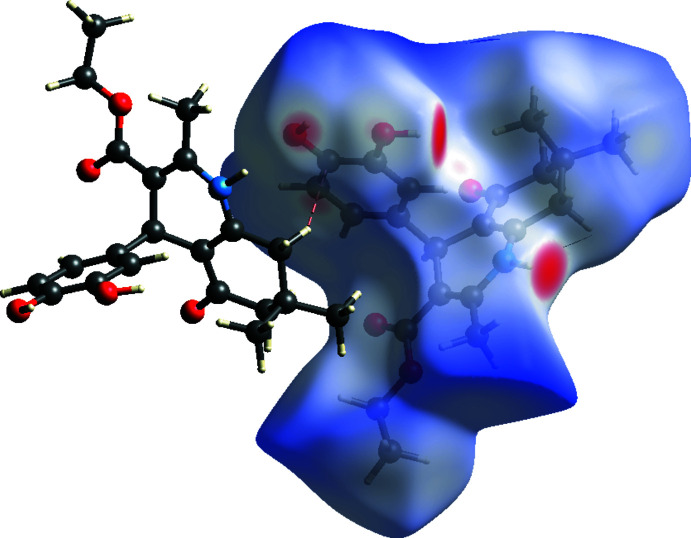
Hirshfeld surface of III mapped over *d*_norm_. Short and long contacts are indicated as red and blue regions, respectively. Contacts with distances approximately equal to the sum of the van der Waals radii are colored white. The close contact between H8*A* and C20 is shown as a dashed line.

**Figure 12:**
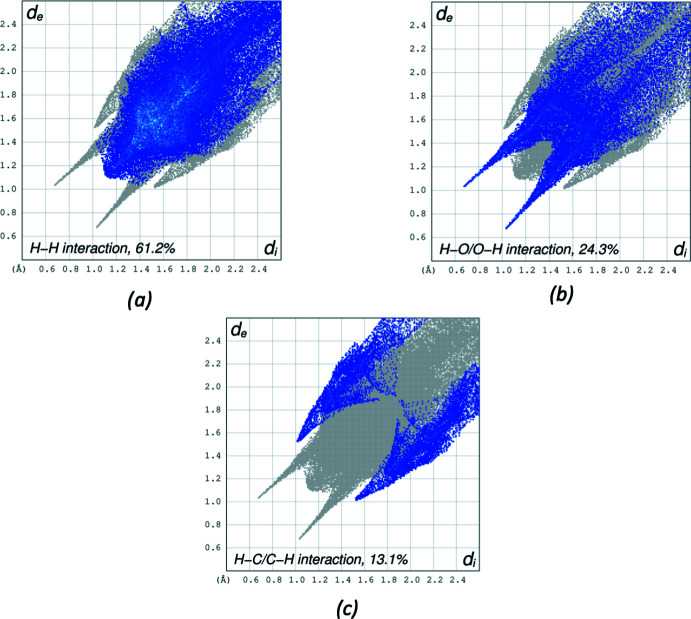
The two-dimensional fingerprint plots for III delineated into (*a*) H⋯H contacts, (*b*) H⋯O/O⋯H contacts, (*c*) H⋯C/C⋯H contacts. Other contact contributions of less than 1% are omitted.

The Hirshfeld surface of the title compound III is mapped over *d*_norm_ in a fixed color scale of −0.7001 (red) to 3.4800 (blue) arbitrary units (Fig. 11 ▸). Besides the obvious short contacts from hydrogen bonds, a short contact of 2.6137 (14) Å between H8*A* and C20 is also observed, indicating a σ-π- inter­action between C8—H8*A* and ring C17–C22. The delineated two-dimensional fingerprint plots shown in Fig. 12 ▸ indicate that two main contributions to the overall Hirshfeld surface area arise from H⋯H contacts (61.2%) and O⋯H/H⋯O contacts (24.3%). C⋯H/H⋯C inter­actions contribute 13.1% of the Hirshfeld surface.

## 5. Database survey

A search for 4-phenyl-5-oxo-hexa­hydro­quinoline-3-carboxyl­ate in the Cambridge Structural Database (CSD version 5.43, November 2021 update; Groom *et al.*, 2016 ▸) resulted in 53 hits, of which a *meta*-methoxyl-substituted 4-phenyl-5-oxo-hexa­hydro­quinoline-3-carboxyl­ate (refcode TANVUC; Li, 2017 ▸) should be mentioned. Similar to the title compounds I and IIA, the *meta*-methoxyl group in TANVUC is *exo* to the 1,4-DHP ring and carbonyl group on the ester is in a *cis* orientation to the endocyclic double bond. All of the resulting hits display common structural features, such as the flat-boat conformation of the 1,4-DHP ring, the envelope conformation of the fused cyclo­hexa­none ring, and the substituted aryl ring at the pseudo-axial position to the 1,4-DHP ring.

## 6. Synthesis and crystallization

An oven-dried 100 ml round-bottom flask equipped with a magnetic stir bar was charged with 10 mmol of dimedone, 10 mmol of ethyl aceto­acetate and 5 mol % of ytterbium(III) tri­fluoro­methane­sulfonate. The mixture was then taken up in 30 ml of absolute ethanol, capped and put under an inert atmosphere of argon, after which the solution was allowed to stir at room temperature for 20 min. the appropriate corres­ponding benzaldehyde (10 mmol) and 10 mmol of ammonium acetate were added to the stirring solution, the solution was allowed to stir at room temperature for 48 h. Reaction progress was monitored *via* TLC. Once the reaction was complete, excess solvent was removed *via* rotary evaporation. The solution was then purified *via* silica column chromatography. The products were crystallized from hexane and ethyl acetate (1:4 *v/v*) as white-to-yellow crystalline solids. Compounds I and III were recrystallized from a minimum of warm methanol, to which hexane was added dropwise to a faint opalescence, and slow evaporation produced diffraction-quality crystals.

Compound I: m.p. 520.5 K. ^1^H NMR: (CDCl_3_) δ ppm 7.28 (*dd*, 1H, *J* = 7.33 and 1.83 Hz); 7.07 (*ddd*, 1H, *J* = 8.24, 7.33 and 1.83 Hz); 6.80 (*d*, 1H, *J* = 7.33 Hz); 6.78 (*d*, 1H, *J* = 8.24 Hz); 5.69 (*s*, *br*, 1H); 5.24 (*s*, 1H); 4.00 (*m*, 2H); 3.78 (*s*, 3H); 2.31 (*d*, 1H, *J* = 23.81 Hz); 2.30 (s, 3H); 2.13 (*q*, 2H, *J* = 16.49 Hz); 2.11 (*d*, 1H, *J* = 32.84 Hz); 1.17 (*t*, 3H, *J* = 7.2 Hz); 1.06 (*s*, 3H); 0.92 (*s*, 3H). ^13^C NMR: (CDCl_3_) δ ppm 195.32; 167.98; 157.61; 148.12; 142.99; 134.51; 131.39; 127.34; 120.06; 111.26; 110.90; 105.26; 59.70; 55.46; 50.80; 41.47; 33.58; 32.69; 29.59; 26.99; 19.53; 14.23. HPLC–MS: calculated for [C_22_H_27_NO_4_+H]^+^ 370.46, observed *m*/*z* 370.1865 ([*M* + 1]^+^, 100% rel. intensity). Compound II: m.p. 517–527 K. ^1^H NMR: (CDCl_3_) δ ppm 7.20 (*d*, 2H, *J* = 9.16 Hz); 6.72 (*d*, 2H, *J* = 9.16 Hz); 5.76 (*s*, *br*, 1H); 4.05 (*q*, 2H, *J* = 7.33 Hz); 3.73 (*s*, 3H); 2.36 (*s*, 3H); 2.32 (*d*, 1H, *J* = 16.03 Hz); 2.22 (*d*, 1H, *J* = 16.03); 2.16 (*t*, 2H, *J* = 17.40 Hz); 1.19 (*t*, 3H, *J* = 7.33 Hz); 1.06 (*s*, 3H); 0.93 (*s*, 3H). ^13^C NMR: (CDCl_3_) δppm 195.58; 167.57; 157.83; 147.56; 143.05; 139.62; 129.06; 113.31; 112.61; 106.47; 59.91; 55.21; 50.79; 41.29; 35.75; 32.84; 29.52; 27.30; 19.61; 14.32. HPLC–MS: calculated for [C_22_H_27_NO_4_+H]^+^ 370.46, observed *m*/*z* 370.1873 ([*M* + 1]^+^, 100% rel. intensity). Compound III: ^1^H NMR: (acetone-*d*_6_) δ ppm 7.97 (*s*, 1H); 7.54 (*s*, 1H); 7.45 (*s*, 1H); 6.77 (*t*, 1H, *J* = 1.14 Hz); 6.59 (*d*, 2H, *J* = 1.14 Hz); 4.88 (*s*, 1H); 4.00 (*q*, 2H, *J* = 7.2 Hz); 2.42 (*d*, 1H, *J* = 16.94 Hz); 2.31 (*s*, 3H); 2.30 (*dd*, 1H, *J* = 16.94 and 1.37 Hz); 2.15 (*d*, 1H, *J* = 16.03 Hz); 2.00 (*dd*, 1H, *J* = 16.03 and 1.37 Hz); 1.16 (*t*, 3H, *J* = 7.2 Hz); 1.02 (*s*, 3H); 0.90 (*s*, 3H). ^13^C NMR: (acetone-*d*_6_) δ ppm 193.99; 167.22; 148.25; 144.26; 144.13; 143.04; 140.03; 119.24; 115.27; 114.44; 111.46; 104.96; 58.99; 50.60; 40.03; 35.49; 32.22; 26.35; 22.47; 17.99; 13.84. HPLC–MS: calculated for [C_21_H_25_NO_5_+H]^+^ 372.43, observed *m*/*z* 372.1657 ([*M* + 1]^+^, 100% rel. intensity).

## 7. Refinement

Crystal data, data collection and structure refinement details are summarized in Table 6 ▸. Carbon-bound hydrogen atoms on all three compounds were fixed geometrically and treated as riding with C—H = 0.95–0.98 Å and refined with *U*_iso_(H) = 1.2*U*_eq_ (CH, CH_2_) or 1.5*U*_eq_ (CH_3_). Hydrogen atoms attached to nitro­gen and oxygen were found in difference-Fourier map and refined freely. Eight reflections (010, 0 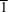 0, 0 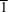 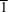 , 011, 00 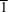 , 001, 002, and 00 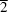 ) in compound I and eight reflections (040, 020, 123, 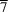 23, 076, 031, 112, and 516) in compound III were omitted because of poor agreement between the observed and calculated intensities.

Data of compound I were acquired at room temperature due to the disintegration of the crystals at low temperatures. The sample measured was identified as two crystals, mis-oriented by 0.24° approximately about the [001] reciprocal-space axis. For the purposes of data collection and subsequent structure refinement, the structure was treated using facilities for handling twinning by non-merohedry, namely HKLF5 data in *SHELXL* (Sheldrick, 2015 ▸), yielding a ratio of 0.866 (2):0.134 (2) for the two crystals. In compound I, the ethyl group on the carb­oxy­lic ester is disordered and was modeled at 50% occupancy at each site. Atomic displacement equivalency restraints and bond-length restraints (Sheldrick, 2015 ▸) were applied to the carbon atoms and the single-bond oxygen atom of the disordered ester group.

The crystals of compound II were found to be pseudo-merohedric twins by a 180° rotation about the *c* axis. Application of the twin operation (−1, 0, 0, 0, −1, 0, 0, 0, 1) yielded a twin component ratio of 0.6938 (8):0.3062 (8). The ester group on mol­ecule *B* is also disordered. Atomic displacement equivalency restraints were applied to the two carbons and the single bond oxygen on the ethyl group. Restraints were applied to bond lengths on the atoms of the ester as well.

Compound III was co-crystallized with hexa­nes. However, being a mixture of disordered hexane isomers, the refinement around the hexa­nes did not give satisfactory results. The *OLEX2 SMTBX* (Rees *et al.*, 2005 ▸) solvent-masking procedure was used to calculate and mask the solvent-accessible void. There are 192 electrons found in a volume of 464 Å^3^ in one void per unit cell. This is consistent with the presence of one C_6_H_14_ mol­ecule per asymmetric unit, which accounts for 200 electrons per unit cell.

## Supplementary Material

Crystal structure: contains datablock(s) I, II, III. DOI: 10.1107/S2056989022009495/jy2021sup1.cif

Structure factors: contains datablock(s) I. DOI: 10.1107/S2056989022009495/jy2021Isup5.hkl

Structure factors: contains datablock(s) II. DOI: 10.1107/S2056989022009495/jy2021IIsup6.hkl

Structure factors: contains datablock(s) III. DOI: 10.1107/S2056989022009495/jy2021IIIsup7.hkl

Supporting information file. DOI: 10.1107/S2056989022009495/jy2021Isup5.cml

Supporting information file. DOI: 10.1107/S2056989022009495/jy2021IIsup6.cml

Supporting information file. DOI: 10.1107/S2056989022009495/jy2021IIIsup7.cml

CCDC references: 2209652, 2209651, 2209650

Additional supporting information:  crystallographic information; 3D view; checkCIF report

## Figures and Tables

**Table 1:** Calculated parameters (Å, °) related to the 1,4-DHP ring

Compound	1,4-DHP mean plane (C2/C3/C10/C9) r.m.s.d	N to ring mean plane distance	C to ring mean plane distance	Phenyl ring to 1,4-DHP mean planes normal-to-normal angle	N1—C4—C17—C18 torsion angle
I	0.015	0.159 (3)	0.341 (3)	89.09 (7)	173.28 (16)
IIA	0.005	0.110 (2)	0.295 (3)	92.52 (6)	1.16 (18)
IIB	0.005	0.110 (3)	0.253 (3)	93.52 (6)	13.41 (14)
III	0.001	0.181 (2)	0.399 (2)	90.59 (5)	18.38 (15)

**Table 2:** Parameters (Å, °) related to the envelope conformation on the cyclo­hexa­none ring

Compound	Mean plane (C5/C6/C8–C10) r.m.s.d	C7 to mean plane distance	C11—C7—C4—C17 torsion angle	Ring puckering parameters		
				*Q*	θ	φ
I	0.025	0.636 (3)	2.53 (18)	0.458 (2)	60.7 (3)	117.2 (3)
IIA	0.015	0.644 (2)	7.96 (14)	0.4616 (18)	56.1 (2)	115.7 (3)
IIB	0.019	0.645 (3)	13.85 (14)	0.4638 (19)	121.2 (2)	303.0 (3)
III	0.028	0.6408 (19)	0.8 (1)	0.4623 (15)	56.53 (19)	111.1 (2)

**Table 3:** Hydrogen-bond geometry (Å, °) for I[Chem scheme1]

*D*—H⋯*A*	*D*—H	H⋯*A*	*D*⋯*A*	*D*—H⋯*A*
N1—H1⋯O1^i^	0.88 (2)	2.01 (2)	2.870 (2)	165 (2)
C13—H13*B*⋯O2	0.96	2.13	2.846 (3)	131
C13—H13*C*⋯O4^i^	0.96	2.59	3.300 (3)	131
C23—H23*A*⋯O2^ii^	0.96	2.57	3.492 (3)	161

Symmetry codes: (i) 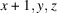 ; (ii) 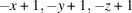 .

**Table 4:** Hydrogen-bond geometry (Å, °) for II[Chem scheme1]

*D*—H⋯*A*	*D*—H	H⋯*A*	*D*⋯*A*	*D*—H⋯*A*
N1*A*—H1*A*⋯O1*B*	0.82 (2)	2.02 (2)	2.827 (2)	167 (2)
N1*B*—H1*B*⋯O1*A* ^i^	0.87 (2)	1.95 (2)	2.8167 (19)	172 (2)

Symmetry code: (i) 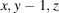 .

**Table 5:** Hydrogen-bond geometry (Å, °) for III[Chem scheme1]

*D*—H⋯*A*	*D*—H	H⋯*A*	*D*⋯*A*	*D*—H⋯*A*
N1—H1⋯O1^i^	0.876 (18)	1.971 (19)	2.8378 (15)	169.8 (16)
O4—H4⋯O2^i^	0.87 (2)	1.82 (2)	2.6894 (14)	175 (2)
O5—H5⋯O1^ii^	0.85 (2)	2.33 (2)	3.0293 (14)	140 (2)

Symmetry codes: (i) 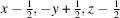 ; (ii) 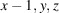 .

**Table 6:** Experimental details

	I	II	III
Crystal data			
Chemical formula	C_22_H_27_NO_4_	C_22_H_27_NO_4_	C_21_H_25_NO_5_
*M*_r_	369.44	369.44	371.42
Crystal system, space group	Triclinic, *P* 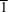	Monoclinic, *P*2_1_/*n*	Monoclinic, *P*2_1_/*n*
Temperature (K)	300	120	100
*a*, *b*, *c* (Å)	7.2941 (2), 9.6773 (3), 14.4302 (4)	15.3492 (15), 14.0314 (14), 18.3862 (18)	9.2745 (3), 22.1655 (7), 11.3475 (4)
α, β, γ (°)	82.1992 (17), 88.3216 (16), 75.9397 (16)	90, 90.0834 (17), 90	90, 108.2014 (17), 90
*V* (Å^3^)	978.92 (5)	3959.8 (7)	2216.03 (13)
*Z*	2	8	4
Radiation type	Mo *K*α	Mo *K*α	Mo *K*α
μ (mm^−1^)	0.09	0.09	0.08
Crystal size (mm)	0.35 × 0.19 × 0.14	0.35 × 0.15 × 0.14	0.64 × 0.13 × 0.06

Data collection			
Diffractometer	Bruker SMART BREEZE CCD	Bruker APEXII CCD	Bruker SMART BREEZE CCD
No. of measured, independent and observed [*I* > 2σ(*I*)] reflections	34490, 34490, 22410	66519, 8085, 7121	40175, 5517, 4263
*R*_int_	–	0.055	0.046
(sin θ/λ)_max_ (Å^−1^)	0.670	0.625	0.668

Refinement			
*R*[*F* ^2^ > 2σ(*F* ^2^)], *wR*(*F* ^2^), *S*	0.055, 0.145, 1.03	0.039, 0.093, 1.04	0.045, 0.123, 1.04
No. of reflections	34490	8085	5517
No. of parameters	285	536	260
No. of restraints	39	39	0
H-atom treatment	H atoms treated by a mixture of independent and constrained refinement	H atoms treated by a mixture of independent and constrained refinement	H atoms treated by a mixture of independent and constrained refinement
Δρ_max_, Δρ_min_ (e Å^−3^)	0.25, −0.17	0.33, −0.30	0.41, −0.21

Computer programs: *APEX2* (Bruker, 2012 ▸), *SAINT* (Bruker, 2016 ▸), *SHELXS* (Sheldrick, 2008 ▸), *SHELXL2018/1* (Sheldrick, 2015 ▸), *OLEX2* (Dolomanov *et al.*, 2009 ▸).

## supplementary crystallographic information

### Ethyl 4-(2-methoxyphenyl)-2,7,7-trimethyl-5-oxo-1,4,5,6,7,8-hexahydroquinoline-3-carboxylate (I) Crystal data

**Table:**

C_22_H_27_NO_4_	*Z* = 2
*M**_r_* = 369.44	*F*(000) = 396
Triclinic, *P*1	*D*_x_ = 1.253 Mg m^−^^3^
*a* = 7.2941 (2) Å	Mo *K*α radiation, λ = 0.71073 Å
*b* = 9.6773 (3) Å	Cell parameters from 7681 reflections
*c* = 14.4302 (4) Å	θ = 2.5–22.8°
α = 82.1992 (17)°	µ = 0.09 mm^−^^1^
β = 88.3216 (16)°	*T* = 300 K
γ = 75.9397 (16)°	Prism, colourless
*V* = 978.92 (5) Å^3^	0.35 × 0.19 × 0.14 mm

### Ethyl 4-(2-methoxyphenyl)-2,7,7-trimethyl-5-oxo-1,4,5,6,7,8-hexahydroquinoline-3-carboxylate (I) Data collection

**Table:**

Bruker SMART BREEZE CCD diffractometer	22410 reflections with *I* > 2σ(*I*)
Radiation source: 2 kW sealed X-ray tube	θ_max_ = 28.4°, θ_min_ = 2.9°
φ and ω scans	*h* = −9→9
34490 measured reflections	*k* = −12→12
34490 independent reflections	*l* = −19→19

### Ethyl 4-(2-methoxyphenyl)-2,7,7-trimethyl-5-oxo-1,4,5,6,7,8-hexahydroquinoline-3-carboxylate (I) Refinement

**Table:**

Refinement on *F*^2^	Primary atom site location: structure-invariant direct methods
Least-squares matrix: full	Hydrogen site location: mixed
*R*[*F*^2^ > 2σ(*F*^2^)] = 0.055	H atoms treated by a mixture of independent and constrained refinement
*wR*(*F*^2^) = 0.145	*w* = 1/[σ^2^(*F*_o_^2^) + (0.0533*P*)^2^ + 0.1957*P*] where *P* = (*F*_o_^2^ + 2*F*_c_^2^)/3
*S* = 1.03	(Δ/σ)_max_ < 0.001
34490 reflections	Δρ_max_ = 0.25 e Å^−^^3^
285 parameters	Δρ_min_ = −0.17 e Å^−^^3^
39 restraints	

### Ethyl 4-(2-methoxyphenyl)-2,7,7-trimethyl-5-oxo-1,4,5,6,7,8-hexahydroquinoline-3-carboxylate (I) Special details

**Table:**

Geometry. All esds (except the esd in the dihedral angle between two l.s. planes) are estimated using the full covariance matrix. The cell esds are taken into account individually in the estimation of esds in distances, angles and torsion angles; correlations between esds in cell parameters are only used when they are defined by crystal symmetry. An approximate (isotropic) treatment of cell esds is used for estimating esds involving l.s. planes.
Refinement. Refined as a 2-component twin. Twin law (-1 0 0 0 -1 0 0.0123 -0.407 1) was applied and the structure was refined using HKLF5 data, yielding a ratio of 0.866 (2):0.134 (2) for the two twin components.

### Ethyl 4-(2-methoxyphenyl)-2,7,7-trimethyl-5-oxo-1,4,5,6,7,8-hexahydroquinoline-3-carboxylate (I) Fractional atomic coordinates and isotropic or equivalent isotropic displacement parameters (Å^2^)

**Table:**

	*x*	*y*	*z*	*U*_iso_*/*U*_eq_	Occ. (<1)
O1	0.25132 (18)	0.32563 (17)	0.92561 (11)	0.0527 (4)	
O2	0.6827 (3)	0.6344 (2)	0.60678 (12)	0.0767 (6)	
O4	0.2571 (2)	0.3067 (2)	0.67486 (12)	0.0678 (5)	
N1	0.8600 (2)	0.39276 (19)	0.87300 (13)	0.0423 (5)	
H1	0.973 (3)	0.384 (2)	0.8957 (16)	0.059 (7)*	
C2	0.8111 (3)	0.4770 (2)	0.78765 (15)	0.0392 (5)	
C3	0.6490 (3)	0.4763 (2)	0.74504 (14)	0.0367 (5)	
C4	0.5269 (3)	0.3751 (2)	0.78681 (13)	0.0353 (5)	
H4	0.393905	0.425869	0.777076	0.042*	
C5	0.4146 (3)	0.3084 (2)	0.95350 (14)	0.0356 (5)	
C6	0.4605 (3)	0.2630 (2)	1.05554 (14)	0.0428 (5)	
H6A	0.385700	0.195992	1.080077	0.051*	
H6B	0.421654	0.346858	1.087912	0.051*	
C7	0.6677 (3)	0.1929 (2)	1.07869 (14)	0.0415 (5)	
C8	0.7858 (3)	0.2895 (2)	1.02836 (14)	0.0415 (5)	
H8A	0.772188	0.373330	1.060330	0.050*	
H8B	0.917788	0.238044	1.031904	0.050*	
C9	0.7317 (3)	0.3380 (2)	0.92783 (14)	0.0343 (5)	
C10	0.5631 (2)	0.3384 (2)	0.89123 (13)	0.0332 (5)	
C11	0.7238 (3)	0.0433 (2)	1.04788 (19)	0.0628 (7)	
H11A	0.646747	−0.015615	1.079713	0.094*	
H11B	0.854309	0.000489	1.063006	0.094*	
H11C	0.705584	0.051153	0.981582	0.094*	
C12	0.7000 (4)	0.1791 (3)	1.18439 (16)	0.0682 (8)	
H12A	0.667684	0.272852	1.204085	0.102*	
H12B	0.830489	0.134601	1.198617	0.102*	
H12C	0.622130	0.121222	1.216662	0.102*	
C13	0.9461 (3)	0.5694 (3)	0.75745 (17)	0.0561 (6)	
H13A	0.928499	0.645503	0.795594	0.084*	
H13B	0.922670	0.609745	0.693126	0.084*	
H13C	1.073547	0.512033	0.764320	0.084*	
C14	0.5891 (3)	0.5687 (2)	0.65630 (15)	0.0449 (5)	
C17	0.5638 (3)	0.2400 (2)	0.73826 (14)	0.0390 (5)	
C18	0.4292 (3)	0.2107 (2)	0.68159 (15)	0.0457 (5)	
C19	0.4721 (4)	0.0891 (3)	0.63638 (17)	0.0579 (7)	
H19	0.382214	0.071800	0.597968	0.069*	
C20	0.6446 (4)	−0.0051 (3)	0.64774 (18)	0.0659 (7)	
H20	0.671714	−0.086456	0.617278	0.079*	
C21	0.7777 (4)	0.0195 (3)	0.70378 (19)	0.0685 (8)	
H21	0.895172	−0.045139	0.712177	0.082*	
C22	0.7362 (3)	0.1416 (2)	0.74789 (16)	0.0532 (6)	
H22	0.828068	0.157980	0.785496	0.064*	
C23	0.1214 (4)	0.2879 (4)	0.61208 (19)	0.0818 (9)	
H23A	0.174511	0.285596	0.550485	0.123*	
H23B	0.011391	0.366392	0.610837	0.123*	
H23C	0.086578	0.199208	0.632473	0.123*	
O3	0.4114 (15)	0.559 (2)	0.6315 (8)	0.048 (2)	0.67 (7)
C15	0.3262 (10)	0.6449 (8)	0.5454 (4)	0.0569 (15)	0.777 (19)
H15A	0.251974	0.591524	0.516476	0.068*	0.777 (19)
H15B	0.425664	0.660874	0.502199	0.068*	0.777 (19)
C16	0.2017 (8)	0.7873 (7)	0.5621 (3)	0.0705 (16)	0.87 (2)
H16A	0.145669	0.838148	0.504055	0.106*	0.87 (2)
H16B	0.275985	0.842813	0.587485	0.106*	0.87 (2)
H16C	0.104003	0.772189	0.605509	0.106*	0.87 (2)
O3A	0.415 (3)	0.601 (5)	0.6407 (19)	0.059 (4)	0.33 (7)
C15A	0.370 (3)	0.698 (3)	0.5526 (14)	0.061 (5)	0.223 (19)
H15C	0.405562	0.787441	0.555021	0.073*	0.223 (19)
H15D	0.432489	0.653296	0.500041	0.073*	0.223 (19)
C16A	0.169 (5)	0.720 (8)	0.546 (3)	0.095 (11)	0.13 (2)
H16D	0.124851	0.778836	0.488685	0.142*	0.13 (2)
H16E	0.110947	0.767237	0.597962	0.142*	0.13 (2)
H16F	0.137650	0.629016	0.548632	0.142*	0.13 (2)

### Ethyl 4-(2-methoxyphenyl)-2,7,7-trimethyl-5-oxo-1,4,5,6,7,8-hexahydroquinoline-3-carboxylate (I) Atomic displacement parameters (Å^2^)

**Table:**

	*U*^11^	*U*^22^	*U*^33^	*U*^12^	*U*^13^	*U*^23^
O1	0.0281 (8)	0.0690 (11)	0.0605 (10)	−0.0152 (7)	−0.0053 (7)	0.0009 (8)
O2	0.0943 (14)	0.0879 (14)	0.0531 (11)	−0.0447 (12)	0.0001 (10)	0.0141 (10)
O4	0.0469 (10)	0.0883 (13)	0.0737 (12)	−0.0127 (9)	−0.0184 (8)	−0.0322 (10)
N1	0.0260 (9)	0.0524 (12)	0.0487 (11)	−0.0131 (8)	−0.0046 (8)	0.0003 (9)
C2	0.0344 (11)	0.0393 (12)	0.0436 (13)	−0.0091 (9)	0.0055 (9)	−0.0055 (10)
C3	0.0365 (11)	0.0347 (11)	0.0378 (12)	−0.0062 (9)	0.0022 (9)	−0.0060 (9)
C4	0.0275 (10)	0.0407 (12)	0.0367 (12)	−0.0062 (8)	−0.0040 (8)	−0.0039 (9)
C5	0.0297 (10)	0.0340 (11)	0.0438 (12)	−0.0080 (8)	−0.0012 (9)	−0.0066 (9)
C6	0.0383 (12)	0.0474 (13)	0.0429 (13)	−0.0111 (10)	0.0027 (10)	−0.0057 (10)
C7	0.0412 (12)	0.0423 (12)	0.0394 (12)	−0.0084 (10)	−0.0043 (9)	−0.0016 (10)
C8	0.0352 (11)	0.0449 (13)	0.0447 (13)	−0.0093 (10)	−0.0094 (9)	−0.0054 (10)
C9	0.0292 (10)	0.0357 (11)	0.0386 (12)	−0.0083 (8)	−0.0011 (9)	−0.0061 (9)
C10	0.0275 (10)	0.0351 (11)	0.0367 (11)	−0.0064 (8)	−0.0034 (8)	−0.0053 (9)
C11	0.0600 (15)	0.0419 (14)	0.0818 (19)	−0.0060 (12)	−0.0071 (14)	−0.0017 (13)
C12	0.0687 (17)	0.090 (2)	0.0428 (15)	−0.0203 (15)	−0.0092 (12)	0.0054 (14)
C13	0.0487 (14)	0.0604 (15)	0.0646 (16)	−0.0262 (12)	0.0055 (12)	−0.0040 (13)
C14	0.0553 (15)	0.0398 (13)	0.0385 (13)	−0.0086 (11)	0.0041 (11)	−0.0074 (10)
C17	0.0411 (12)	0.0416 (12)	0.0352 (12)	−0.0130 (10)	−0.0012 (9)	−0.0027 (10)
C18	0.0457 (13)	0.0524 (14)	0.0432 (13)	−0.0196 (11)	−0.0011 (10)	−0.0066 (11)
C19	0.0748 (18)	0.0606 (16)	0.0493 (15)	−0.0332 (15)	−0.0029 (13)	−0.0142 (13)
C20	0.095 (2)	0.0472 (15)	0.0603 (17)	−0.0204 (15)	0.0043 (15)	−0.0175 (13)
C21	0.0742 (18)	0.0518 (16)	0.0733 (19)	0.0020 (13)	−0.0064 (15)	−0.0167 (14)
C22	0.0511 (14)	0.0502 (14)	0.0563 (15)	−0.0044 (11)	−0.0088 (11)	−0.0130 (12)
C23	0.0526 (15)	0.135 (3)	0.0679 (19)	−0.0298 (17)	−0.0130 (14)	−0.0334 (18)
O3	0.052 (2)	0.048 (5)	0.042 (2)	−0.010 (2)	−0.0128 (16)	0.003 (3)
C15	0.067 (3)	0.060 (3)	0.041 (2)	−0.015 (2)	−0.0144 (19)	0.003 (2)
C16	0.083 (3)	0.058 (3)	0.063 (3)	−0.009 (2)	−0.0144 (19)	0.006 (2)
O3A	0.062 (5)	0.057 (9)	0.044 (5)	0.004 (6)	−0.010 (4)	0.008 (6)
C15A	0.077 (9)	0.057 (10)	0.038 (7)	−0.005 (8)	−0.009 (6)	0.012 (7)
C16A	0.104 (19)	0.10 (2)	0.074 (16)	−0.016 (18)	−0.034 (14)	0.016 (18)

### Ethyl 4-(2-methoxyphenyl)-2,7,7-trimethyl-5-oxo-1,4,5,6,7,8-hexahydroquinoline-3-carboxylate (I) Geometric parameters (Å, º)

**Table:**

O1—C5	1.233 (2)	C13—H13A	0.9600
O2—C14	1.200 (2)	C13—H13B	0.9600
O4—C18	1.366 (3)	C13—H13C	0.9600
O4—C23	1.420 (3)	C14—O3	1.382 (10)
N1—H1	0.88 (2)	C14—O3A	1.25 (3)
N1—C2	1.386 (3)	C17—C18	1.399 (3)
N1—C9	1.366 (2)	C17—C22	1.378 (3)
C2—C3	1.350 (3)	C18—C19	1.388 (3)
C2—C13	1.502 (3)	C19—H19	0.9300
C3—C4	1.532 (3)	C19—C20	1.362 (3)
C3—C14	1.471 (3)	C20—H20	0.9300
C4—H4	0.9800	C20—C21	1.366 (3)
C4—C10	1.516 (3)	C21—H21	0.9300
C4—C17	1.529 (3)	C21—C22	1.383 (3)
C5—C6	1.502 (3)	C22—H22	0.9300
C5—C10	1.446 (3)	C23—H23A	0.9600
C6—H6A	0.9700	C23—H23B	0.9600
C6—H6B	0.9700	C23—H23C	0.9600
C6—C7	1.525 (3)	O3—C15	1.460 (10)
C7—C8	1.521 (3)	C15—H15A	0.9700
C7—C11	1.528 (3)	C15—H15B	0.9700
C7—C12	1.533 (3)	C15—C16	1.501 (8)
C8—H8A	0.9700	C16—H16A	0.9600
C8—H8B	0.9700	C16—H16B	0.9600
C8—C9	1.496 (3)	C16—H16C	0.9600
C9—C10	1.351 (2)	O3A—C15A	1.47 (2)
C11—H11A	0.9600	C15A—H15C	0.9700
C11—H11B	0.9600	C15A—H15D	0.9700
C11—H11C	0.9600	C15A—C16A	1.43 (4)
C12—H12A	0.9600	C16A—H16D	0.9600
C12—H12B	0.9600	C16A—H16E	0.9600
C12—H12C	0.9600	C16A—H16F	0.9600
			
C18—O4—C23	118.3 (2)	H13A—C13—H13B	109.5
C2—N1—H1	118.3 (15)	H13A—C13—H13C	109.5
C9—N1—H1	118.9 (15)	H13B—C13—H13C	109.5
C9—N1—C2	122.25 (17)	O2—C14—C3	126.6 (2)
N1—C2—C13	112.68 (18)	O2—C14—O3	123.1 (5)
C3—C2—N1	119.51 (18)	O2—C14—O3A	116.9 (14)
C3—C2—C13	127.6 (2)	O3—C14—C3	110.0 (5)
C2—C3—C4	120.45 (17)	O3A—C14—C3	115.2 (11)
C2—C3—C14	120.91 (19)	C18—C17—C4	122.77 (19)
C14—C3—C4	118.61 (18)	C22—C17—C4	120.36 (18)
C3—C4—H4	108.2	C22—C17—C18	116.9 (2)
C10—C4—C3	109.37 (15)	O4—C18—C17	116.32 (19)
C10—C4—H4	108.2	O4—C18—C19	123.2 (2)
C10—C4—C17	111.63 (16)	C19—C18—C17	120.5 (2)
C17—C4—C3	111.17 (16)	C18—C19—H19	119.7
C17—C4—H4	108.2	C20—C19—C18	120.6 (2)
O1—C5—C6	119.83 (18)	C20—C19—H19	119.7
O1—C5—C10	121.75 (18)	C19—C20—H20	119.9
C10—C5—C6	118.34 (16)	C19—C20—C21	120.2 (2)
C5—C6—H6A	108.4	C21—C20—H20	119.9
C5—C6—H6B	108.4	C20—C21—H21	120.3
C5—C6—C7	115.67 (17)	C20—C21—C22	119.3 (2)
H6A—C6—H6B	107.4	C22—C21—H21	120.3
C7—C6—H6A	108.4	C17—C22—C21	122.5 (2)
C7—C6—H6B	108.4	C17—C22—H22	118.7
C6—C7—C11	110.41 (18)	C21—C22—H22	118.7
C6—C7—C12	109.80 (18)	O4—C23—H23A	109.5
C8—C7—C6	107.87 (16)	O4—C23—H23B	109.5
C8—C7—C11	110.68 (19)	O4—C23—H23C	109.5
C8—C7—C12	109.10 (18)	H23A—C23—H23B	109.5
C11—C7—C12	108.95 (19)	H23A—C23—H23C	109.5
C7—C8—H8A	108.9	H23B—C23—H23C	109.5
C7—C8—H8B	108.9	C14—O3—C15	118.3 (9)
H8A—C8—H8B	107.7	O3—C15—H15A	109.1
C9—C8—C7	113.37 (16)	O3—C15—H15B	109.1
C9—C8—H8A	108.9	O3—C15—C16	112.5 (9)
C9—C8—H8B	108.9	H15A—C15—H15B	107.8
N1—C9—C8	116.06 (17)	C16—C15—H15A	109.1
C10—C9—N1	119.62 (18)	C16—C15—H15B	109.1
C10—C9—C8	124.19 (18)	C15—C16—H16A	109.5
C5—C10—C4	120.18 (16)	C15—C16—H16B	109.5
C9—C10—C4	120.76 (17)	C15—C16—H16C	109.5
C9—C10—C5	119.03 (18)	H16A—C16—H16B	109.5
C7—C11—H11A	109.5	H16A—C16—H16C	109.5
C7—C11—H11B	109.5	H16B—C16—H16C	109.5
C7—C11—H11C	109.5	C14—O3A—C15A	111 (2)
H11A—C11—H11B	109.5	O3A—C15A—H15C	111.3
H11A—C11—H11C	109.5	O3A—C15A—H15D	111.3
H11B—C11—H11C	109.5	H15C—C15A—H15D	109.2
C7—C12—H12A	109.5	C16A—C15A—O3A	102 (2)
C7—C12—H12B	109.5	C16A—C15A—H15C	111.3
C7—C12—H12C	109.5	C16A—C15A—H15D	111.3
H12A—C12—H12B	109.5	C15A—C16A—H16D	109.5
H12A—C12—H12C	109.5	C15A—C16A—H16E	109.5
H12B—C12—H12C	109.5	C15A—C16A—H16F	109.5
C2—C13—H13A	109.5	H16D—C16A—H16E	109.5
C2—C13—H13B	109.5	H16D—C16A—H16F	109.5
C2—C13—H13C	109.5	H16E—C16A—H16F	109.5
			
O1—C5—C6—C7	158.59 (18)	C6—C5—C10—C4	175.97 (18)
O1—C5—C10—C4	−7.4 (3)	C6—C5—C10—C9	−6.2 (3)
O1—C5—C10—C9	170.39 (19)	C6—C7—C8—C9	−47.3 (2)
O2—C14—O3—C15	−6 (2)	C7—C8—C9—N1	−163.74 (17)
O2—C14—O3A—C15A	10 (4)	C7—C8—C9—C10	20.4 (3)
O4—C18—C19—C20	−178.1 (2)	C8—C9—C10—C4	−173.83 (18)
N1—C2—C3—C4	−4.4 (3)	C8—C9—C10—C5	8.4 (3)
N1—C2—C3—C14	177.57 (19)	C9—N1—C2—C3	−17.4 (3)
N1—C9—C10—C4	10.5 (3)	C9—N1—C2—C13	158.14 (19)
N1—C9—C10—C5	−167.32 (18)	C10—C4—C17—C18	125.8 (2)
C2—N1—C9—C8	−161.64 (19)	C10—C4—C17—C22	−55.3 (2)
C2—N1—C9—C10	14.4 (3)	C10—C5—C6—C7	−24.7 (3)
C2—C3—C4—C10	25.1 (3)	C11—C7—C8—C9	73.6 (2)
C2—C3—C4—C17	−98.6 (2)	C12—C7—C8—C9	−166.52 (19)
C2—C3—C14—O2	11.7 (3)	C13—C2—C3—C4	−179.3 (2)
C2—C3—C14—O3	−174.2 (9)	C13—C2—C3—C14	2.7 (3)
C2—C3—C14—O3A	−155 (3)	C14—C3—C4—C10	−156.85 (17)
C3—C4—C10—C5	149.48 (17)	C14—C3—C4—C17	79.4 (2)
C3—C4—C10—C9	−28.3 (2)	C14—O3—C15—C16	−93.5 (15)
C3—C4—C17—C18	−111.8 (2)	C14—O3A—C15A—C16A	179 (5)
C3—C4—C17—C22	67.1 (2)	C17—C4—C10—C5	−87.1 (2)
C3—C14—O3—C15	179.4 (11)	C17—C4—C10—C9	95.2 (2)
C3—C14—O3A—C15A	178 (2)	C17—C18—C19—C20	1.2 (3)
C4—C3—C14—O2	−166.3 (2)	C18—C17—C22—C21	0.4 (3)
C4—C3—C14—O3	7.7 (9)	C18—C19—C20—C21	−0.2 (4)
C4—C3—C14—O3A	27 (3)	C19—C20—C21—C22	−0.7 (4)
C4—C17—C18—O4	−3.0 (3)	C20—C21—C22—C17	0.5 (4)
C4—C17—C18—C19	177.7 (2)	C22—C17—C18—O4	178.08 (19)
C4—C17—C22—C21	−178.6 (2)	C22—C17—C18—C19	−1.3 (3)
C5—C6—C7—C8	50.3 (2)	C23—O4—C18—C17	174.8 (2)
C5—C6—C7—C11	−70.8 (2)	C23—O4—C18—C19	−5.8 (3)
C5—C6—C7—C12	169.07 (19)		

### Ethyl 4-(2-methoxyphenyl)-2,7,7-trimethyl-5-oxo-1,4,5,6,7,8-hexahydroquinoline-3-carboxylate (I) Hydrogen-bond geometry (Å, º)

**Table:**

*D*—H···*A*	*D*—H	H···*A*	*D*···*A*	*D*—H···*A*
N1—H1···O1^i^	0.88 (2)	2.01 (2)	2.870 (2)	165 (2)
C13—H13*B*···O2	0.96	2.13	2.846 (3)	131
C13—H13*C*···O4^i^	0.96	2.59	3.300 (3)	131
C23—H23*A*···O2^ii^	0.96	2.57	3.492 (3)	161

Symmetry codes: (i) *x*+1, *y*, *z*; (ii) −*x*+1, −*y*+1, −*z*+1.

### Ethyl 4-(4-methoxyphenyl)-2,7,7-trimethyl-5-oxo-1,4,5,6,7,8-hexahydroquinoline-3-carboxylate (II) Crystal data

**Table:**

C_22_H_27_NO_4_	*F*(000) = 1584
*M**_r_* = 369.44	*D*_x_ = 1.239 Mg m^−^^3^
Monoclinic, *P*2_1_/*n*	Mo *K*α radiation, λ = 0.71073 Å
*a* = 15.3492 (15) Å	Cell parameters from 9980 reflections
*b* = 14.0314 (14) Å	θ = 2.3–26.4°
*c* = 18.3862 (18) Å	µ = 0.09 mm^−^^1^
β = 90.0834 (17)°	*T* = 120 K
*V* = 3959.8 (7) Å^3^	Rod, colourless
*Z* = 8	0.35 × 0.15 × 0.14 mm

### Ethyl 4-(4-methoxyphenyl)-2,7,7-trimethyl-5-oxo-1,4,5,6,7,8-hexahydroquinoline-3-carboxylate (II) Data collection

**Table:**

Bruker APEXII CCD diffractometer	*R*_int_ = 0.055
φ and ω scans	θ_max_ = 26.4°, θ_min_ = 1.3°
66519 measured reflections	*h* = −19→19
8085 independent reflections	*k* = −17→17
7121 reflections with *I* > 2σ(*I*)	*l* = −22→22

### Ethyl 4-(4-methoxyphenyl)-2,7,7-trimethyl-5-oxo-1,4,5,6,7,8-hexahydroquinoline-3-carboxylate (II) Refinement

**Table:**

Refinement on *F*^2^	Primary atom site location: structure-invariant direct methods
Least-squares matrix: full	Hydrogen site location: mixed
*R*[*F*^2^ > 2σ(*F*^2^)] = 0.039	H atoms treated by a mixture of independent and constrained refinement
*wR*(*F*^2^) = 0.093	*w* = 1/[σ^2^(*F*_o_^2^) + (0.0433*P*)^2^ + 1.0284*P*] where *P* = (*F*_o_^2^ + 2*F*_c_^2^)/3
*S* = 1.04	(Δ/σ)_max_ = 0.001
8085 reflections	Δρ_max_ = 0.33 e Å^−^^3^
536 parameters	Δρ_min_ = −0.30 e Å^−^^3^
39 restraints	

### Ethyl 4-(4-methoxyphenyl)-2,7,7-trimethyl-5-oxo-1,4,5,6,7,8-hexahydroquinoline-3-carboxylate (II) Special details

**Table:**

Geometry. All esds (except the esd in the dihedral angle between two l.s. planes) are estimated using the full covariance matrix. The cell esds are taken into account individually in the estimation of esds in distances, angles and torsion angles; correlations between esds in cell parameters are only used when they are defined by crystal symmetry. An approximate (isotropic) treatment of cell esds is used for estimating esds involving l.s. planes.
Refinement. Refined as a 2-component twin. Application of the twin law (-1, 0, 0, 0, -1, 0, 0, 0, 1) yielded a twin domain ratio of 0.6938 (8):0.3062 (8).

### Ethyl 4-(4-methoxyphenyl)-2,7,7-trimethyl-5-oxo-1,4,5,6,7,8-hexahydroquinoline-3-carboxylate (II) Fractional atomic coordinates and isotropic or equivalent isotropic displacement parameters (Å^2^)

**Table:**

	*x*	*y*	*z*	*U*_iso_*/*U*_eq_	Occ. (<1)
O1A	0.45938 (8)	1.14027 (8)	0.37758 (7)	0.0261 (3)	
O2A	0.83266 (9)	0.94867 (10)	0.38491 (8)	0.0353 (3)	
O3A	0.76656 (8)	1.07646 (9)	0.33709 (7)	0.0239 (3)	
O4A	0.58302 (9)	1.01706 (9)	0.04318 (7)	0.0279 (3)	
N1A	0.57551 (10)	0.83505 (11)	0.40985 (8)	0.0195 (3)	
H1A	0.5659 (13)	0.7789 (15)	0.4186 (11)	0.023 (5)*	
C2A	0.66070 (11)	0.86446 (12)	0.39877 (9)	0.0196 (4)	
C3A	0.67643 (11)	0.95409 (12)	0.37482 (9)	0.0186 (4)	
C4A	0.60238 (10)	1.01965 (12)	0.35175 (9)	0.0175 (3)	
H4A	0.616041	1.085397	0.369398	0.021*	
C5A	0.44879 (11)	1.05602 (12)	0.39480 (9)	0.0182 (4)	
C6A	0.36366 (11)	1.02348 (12)	0.42708 (10)	0.0200 (4)	
H6AA	0.315982	1.062202	0.406008	0.024*	
H6AB	0.364850	1.035734	0.480100	0.024*	
C7A	0.34314 (11)	0.91751 (12)	0.41443 (9)	0.0193 (4)	
C8A	0.42224 (11)	0.85979 (12)	0.43937 (9)	0.0186 (3)	
H8AA	0.424860	0.860609	0.493173	0.022*	
H8AB	0.414748	0.792748	0.423785	0.022*	
C9A	0.50663 (11)	0.89679 (12)	0.40982 (9)	0.0170 (3)	
C10A	0.51830 (11)	0.98787 (12)	0.38714 (9)	0.0167 (3)	
C11A	0.32322 (12)	0.90078 (13)	0.33413 (10)	0.0248 (4)	
H11D	0.275190	0.942331	0.318973	0.037*	
H11E	0.306604	0.834047	0.326715	0.037*	
H11F	0.375071	0.915231	0.305108	0.037*	
C12A	0.26398 (12)	0.88893 (13)	0.45999 (11)	0.0266 (4)	
H12D	0.276470	0.900163	0.511574	0.040*	
H12E	0.251289	0.821217	0.452347	0.040*	
H12F	0.213502	0.927113	0.445243	0.040*	
C13A	0.72712 (12)	0.79058 (13)	0.41851 (11)	0.0263 (4)	
H13D	0.752914	0.806281	0.465804	0.039*	
H13E	0.772869	0.789171	0.381402	0.039*	
H13F	0.699027	0.727966	0.421239	0.039*	
C14A	0.76640 (11)	0.98918 (13)	0.36709 (9)	0.0214 (4)	
C15A	0.85044 (12)	1.11883 (14)	0.32108 (11)	0.0289 (4)	
H15E	0.893385	1.068708	0.308859	0.035*	
H15F	0.872177	1.154790	0.363730	0.035*	
C16A	0.83761 (13)	1.18449 (14)	0.25765 (12)	0.0352 (5)	
H16G	0.811091	1.149255	0.217271	0.053*	
H16H	0.894095	1.210040	0.242322	0.053*	
H16I	0.799255	1.237092	0.271891	0.053*	
C17A	0.59364 (10)	1.02320 (12)	0.26930 (9)	0.0177 (4)	
C18A	0.57574 (11)	0.93988 (12)	0.23039 (9)	0.0214 (4)	
H18A	0.565952	0.882052	0.255969	0.026*	
C19A	0.57207 (11)	0.94030 (13)	0.15539 (10)	0.0218 (4)	
H19A	0.560018	0.882987	0.129776	0.026*	
C20A	0.58598 (11)	1.02449 (13)	0.11729 (9)	0.0217 (4)	
C21A	0.60189 (11)	1.10830 (13)	0.15485 (10)	0.0232 (4)	
H21A	0.610207	1.166456	0.129332	0.028*	
C22A	0.60553 (11)	1.10619 (12)	0.23063 (10)	0.0216 (4)	
H22A	0.616536	1.163699	0.256321	0.026*	
C23A	0.58707 (15)	1.10334 (15)	0.00256 (11)	0.0359 (5)	
H23D	0.581901	1.088987	−0.049428	0.054*	
H23E	0.642866	1.135061	0.011879	0.054*	
H23F	0.539242	1.145358	0.017281	0.054*	
O1B	0.53248 (9)	0.63931 (8)	0.41653 (7)	0.0275 (3)	
O2B	0.16407 (8)	0.44710 (11)	0.34002 (8)	0.0367 (3)	
O4B	0.45174 (10)	0.69033 (9)	0.07234 (7)	0.0325 (3)	
N1B	0.41867 (10)	0.33580 (11)	0.37064 (9)	0.0232 (3)	
H1B	0.4275 (14)	0.2749 (16)	0.3763 (12)	0.037 (6)*	
C2B	0.33400 (11)	0.36815 (12)	0.36142 (10)	0.0224 (4)	
C3B	0.31849 (11)	0.46259 (13)	0.35623 (10)	0.0226 (4)	
C4B	0.39216 (11)	0.53471 (12)	0.35400 (10)	0.0201 (4)	
H4B	0.375782	0.589307	0.386149	0.024*	
C5B	0.54390 (12)	0.55231 (12)	0.41031 (9)	0.0207 (4)	
C6B	0.62933 (12)	0.50822 (13)	0.43392 (10)	0.0236 (4)	
H6BA	0.629257	0.502553	0.487587	0.028*	
H6BB	0.677155	0.552131	0.420484	0.028*	
C7B	0.64891 (12)	0.41011 (13)	0.40149 (10)	0.0223 (4)	
C8B	0.56843 (11)	0.34797 (12)	0.41231 (10)	0.0221 (4)	
H8BA	0.576211	0.287574	0.385247	0.026*	
H8BB	0.562928	0.332075	0.464561	0.026*	
C9B	0.48608 (11)	0.39520 (12)	0.38717 (10)	0.0195 (4)	
C10B	0.47532 (11)	0.49112 (12)	0.38389 (10)	0.0190 (4)	
C11B	0.67057 (13)	0.41894 (14)	0.32069 (10)	0.0290 (4)	
H11A	0.681145	0.355422	0.300374	0.044*	
H11B	0.722849	0.458254	0.314753	0.044*	
H11C	0.621608	0.448806	0.295120	0.044*	
C12B	0.72593 (12)	0.36470 (15)	0.44191 (12)	0.0314 (5)	
H12A	0.711607	0.358440	0.493620	0.047*	
H12B	0.777587	0.405106	0.436484	0.047*	
H12C	0.737796	0.301528	0.421460	0.047*	
C13B	0.26813 (12)	0.28863 (14)	0.35968 (12)	0.0323 (5)	
H13A	0.297954	0.227423	0.365980	0.048*	
H13B	0.237582	0.289167	0.312845	0.048*	
H13C	0.226000	0.297564	0.399095	0.048*	
C14B	0.22780 (13)	0.49593 (14)	0.34891 (12)	0.0317 (4)	
C17B	0.40645 (11)	0.57381 (12)	0.27734 (10)	0.0204 (4)	
C18B	0.41311 (12)	0.51382 (13)	0.21751 (10)	0.0235 (4)	
H18B	0.407494	0.446967	0.224204	0.028*	
C19B	0.42785 (12)	0.54948 (13)	0.14796 (10)	0.0247 (4)	
H19B	0.432202	0.507346	0.107685	0.030*	
C20B	0.43611 (11)	0.64698 (13)	0.13795 (10)	0.0231 (4)	
C21B	0.42916 (11)	0.70792 (12)	0.19706 (10)	0.0231 (4)	
H21B	0.434268	0.774830	0.190398	0.028*	
C22B	0.41482 (12)	0.67106 (12)	0.26552 (10)	0.0220 (4)	
H22B	0.410556	0.713382	0.305688	0.026*	
C23B	0.46826 (19)	0.62980 (17)	0.01175 (12)	0.0469 (6)	
H23A	0.481787	0.668742	−0.031018	0.070*	
H23B	0.416580	0.590883	0.001799	0.070*	
H23C	0.517779	0.588112	0.022755	0.070*	
O3B	0.2269 (3)	0.5956 (3)	0.3723 (4)	0.0335 (6)	0.432 (8)
C15B	0.1402 (3)	0.6417 (4)	0.3742 (3)	0.0372 (7)	0.465 (5)
H15A	0.138304	0.689695	0.413646	0.045*	0.465 (5)
H15B	0.094562	0.593444	0.383626	0.045*	0.465 (5)
C16B	0.1245 (4)	0.6886 (5)	0.3024 (3)	0.0467 (11)	0.465 (5)
H16A	0.123933	0.640269	0.263942	0.070*	0.465 (5)
H16B	0.171021	0.734776	0.292792	0.070*	0.465 (5)
H16C	0.068246	0.721642	0.303351	0.070*	0.465 (5)
O3C	0.21906 (19)	0.5876 (2)	0.3397 (3)	0.0334 (5)	0.568 (8)
C15C	0.1290 (2)	0.6186 (3)	0.3293 (3)	0.0363 (7)	0.535 (5)
H15C	0.107508	0.596737	0.281287	0.044*	0.535 (5)
H15D	0.091485	0.590425	0.367375	0.044*	0.535 (5)
C16C	0.1252 (3)	0.7220 (3)	0.3332 (3)	0.0423 (10)	0.535 (5)
H16D	0.164743	0.749457	0.297027	0.063*	0.535 (5)
H16E	0.142609	0.742950	0.381911	0.063*	0.535 (5)
H16F	0.065517	0.743317	0.323217	0.063*	0.535 (5)

### Ethyl 4-(4-methoxyphenyl)-2,7,7-trimethyl-5-oxo-1,4,5,6,7,8-hexahydroquinoline-3-carboxylate (II) Atomic displacement parameters (Å^2^)

**Table:**

	*U*^11^	*U*^22^	*U*^33^	*U*^12^	*U*^13^	*U*^23^
O1A	0.0244 (6)	0.0130 (6)	0.0411 (8)	0.0011 (5)	0.0035 (6)	0.0025 (5)
O2A	0.0192 (7)	0.0410 (8)	0.0458 (9)	0.0025 (6)	−0.0023 (6)	0.0152 (7)
O3A	0.0159 (6)	0.0237 (7)	0.0323 (7)	−0.0027 (5)	0.0006 (5)	0.0033 (5)
O4A	0.0322 (7)	0.0335 (7)	0.0180 (7)	−0.0002 (6)	−0.0006 (5)	0.0040 (5)
N1A	0.0205 (7)	0.0116 (7)	0.0264 (8)	0.0011 (6)	−0.0005 (6)	0.0012 (6)
C2A	0.0206 (9)	0.0202 (9)	0.0180 (9)	0.0027 (7)	−0.0015 (7)	−0.0033 (7)
C3A	0.0174 (8)	0.0211 (9)	0.0172 (8)	0.0029 (7)	−0.0001 (7)	−0.0001 (7)
C4A	0.0176 (8)	0.0147 (8)	0.0201 (9)	−0.0010 (6)	−0.0002 (7)	−0.0001 (7)
C5A	0.0211 (9)	0.0153 (8)	0.0182 (8)	−0.0009 (7)	−0.0017 (7)	−0.0008 (7)
C6A	0.0211 (9)	0.0169 (9)	0.0221 (9)	0.0026 (7)	0.0024 (7)	−0.0022 (7)
C7A	0.0186 (8)	0.0176 (8)	0.0216 (9)	−0.0006 (7)	0.0004 (7)	0.0010 (7)
C8A	0.0219 (9)	0.0146 (8)	0.0194 (9)	−0.0019 (7)	0.0000 (7)	−0.0005 (6)
C9A	0.0210 (8)	0.0145 (8)	0.0156 (8)	0.0015 (7)	−0.0021 (7)	−0.0002 (7)
C10A	0.0181 (8)	0.0160 (8)	0.0161 (8)	0.0009 (7)	−0.0015 (7)	−0.0010 (7)
C11A	0.0224 (9)	0.0259 (10)	0.0260 (10)	0.0016 (7)	−0.0045 (7)	−0.0046 (8)
C12A	0.0227 (9)	0.0234 (10)	0.0337 (11)	−0.0014 (8)	0.0040 (8)	0.0002 (8)
C13A	0.0242 (9)	0.0220 (9)	0.0325 (11)	0.0054 (7)	−0.0024 (8)	0.0009 (8)
C14A	0.0212 (9)	0.0249 (9)	0.0180 (9)	0.0015 (7)	−0.0019 (7)	−0.0001 (7)
C15A	0.0172 (9)	0.0315 (10)	0.0380 (11)	−0.0060 (8)	0.0015 (8)	−0.0021 (9)
C16A	0.0258 (10)	0.0281 (11)	0.0518 (13)	−0.0037 (8)	0.0069 (9)	0.0071 (10)
C17A	0.0137 (8)	0.0190 (9)	0.0204 (9)	0.0022 (7)	0.0005 (7)	0.0008 (7)
C18A	0.0221 (9)	0.0171 (9)	0.0249 (9)	0.0007 (7)	0.0005 (7)	0.0027 (7)
C19A	0.0215 (9)	0.0202 (9)	0.0236 (9)	0.0013 (7)	−0.0013 (7)	−0.0018 (7)
C20A	0.0162 (8)	0.0305 (10)	0.0183 (9)	−0.0004 (7)	0.0003 (7)	0.0035 (7)
C21A	0.0209 (9)	0.0232 (9)	0.0253 (10)	−0.0046 (7)	−0.0007 (7)	0.0066 (8)
C22A	0.0210 (9)	0.0199 (9)	0.0240 (9)	−0.0032 (7)	−0.0016 (7)	0.0012 (7)
C23A	0.0439 (12)	0.0408 (12)	0.0230 (10)	−0.0123 (10)	0.0000 (9)	0.0099 (9)
O1B	0.0316 (7)	0.0147 (6)	0.0361 (8)	−0.0032 (5)	0.0028 (6)	−0.0020 (6)
O2B	0.0181 (7)	0.0555 (10)	0.0366 (8)	−0.0001 (7)	−0.0001 (6)	0.0014 (7)
O4B	0.0461 (9)	0.0282 (7)	0.0234 (7)	−0.0002 (6)	0.0002 (6)	0.0053 (6)
N1B	0.0199 (7)	0.0121 (7)	0.0378 (9)	0.0002 (6)	−0.0020 (7)	0.0013 (7)
C2B	0.0194 (9)	0.0217 (9)	0.0261 (9)	−0.0015 (7)	−0.0002 (7)	0.0032 (7)
C3B	0.0192 (9)	0.0240 (9)	0.0246 (9)	0.0010 (7)	0.0029 (7)	0.0044 (8)
C4B	0.0193 (9)	0.0153 (8)	0.0258 (9)	0.0034 (7)	0.0029 (7)	0.0006 (7)
C5B	0.0224 (9)	0.0187 (9)	0.0209 (9)	−0.0017 (7)	0.0041 (7)	0.0009 (7)
C6B	0.0225 (9)	0.0221 (9)	0.0262 (10)	−0.0057 (7)	−0.0002 (7)	0.0008 (7)
C7B	0.0193 (9)	0.0212 (9)	0.0265 (10)	0.0011 (7)	0.0018 (7)	0.0042 (7)
C8B	0.0206 (9)	0.0167 (8)	0.0288 (10)	0.0017 (7)	0.0009 (7)	0.0028 (7)
C9B	0.0188 (8)	0.0180 (9)	0.0218 (9)	0.0015 (7)	0.0015 (7)	0.0012 (7)
C10B	0.0200 (9)	0.0150 (8)	0.0220 (9)	0.0002 (7)	0.0030 (7)	0.0004 (7)
C11B	0.0278 (10)	0.0287 (10)	0.0306 (11)	0.0054 (8)	0.0068 (8)	0.0029 (8)
C12B	0.0215 (10)	0.0331 (11)	0.0396 (12)	0.0022 (8)	0.0004 (8)	0.0102 (9)
C13B	0.0238 (10)	0.0266 (10)	0.0464 (12)	−0.0043 (8)	−0.0029 (9)	0.0054 (9)
C14B	0.0222 (7)	0.0316 (8)	0.0411 (10)	0.0077 (6)	0.0042 (7)	0.0096 (8)
C17B	0.0158 (8)	0.0191 (9)	0.0263 (9)	0.0024 (7)	0.0013 (7)	0.0025 (7)
C18B	0.0227 (9)	0.0160 (9)	0.0319 (10)	−0.0016 (7)	−0.0005 (8)	0.0006 (7)
C19B	0.0239 (9)	0.0238 (9)	0.0263 (10)	0.0000 (7)	−0.0022 (8)	−0.0026 (8)
C20B	0.0197 (9)	0.0244 (9)	0.0252 (9)	0.0003 (7)	−0.0018 (7)	0.0046 (7)
C21B	0.0227 (9)	0.0154 (8)	0.0313 (10)	0.0008 (7)	−0.0008 (8)	0.0031 (7)
C22B	0.0217 (9)	0.0159 (8)	0.0285 (10)	0.0047 (7)	−0.0002 (7)	−0.0003 (7)
C23B	0.0762 (18)	0.0404 (13)	0.0241 (11)	−0.0068 (12)	0.0019 (11)	0.0017 (10)
O3B	0.0235 (9)	0.0329 (10)	0.0440 (13)	0.0081 (9)	0.0033 (10)	0.0089 (11)
C15B	0.0271 (12)	0.0375 (13)	0.0470 (15)	0.0070 (11)	0.0016 (12)	0.0084 (13)
C16B	0.0342 (18)	0.051 (2)	0.055 (2)	0.0052 (18)	0.0014 (18)	0.0084 (19)
O3C	0.0231 (8)	0.0314 (9)	0.0456 (12)	0.0106 (7)	0.0001 (9)	0.0067 (10)
C15C	0.0255 (11)	0.0327 (12)	0.0507 (15)	0.0126 (10)	−0.0021 (11)	0.0033 (11)
C16C	0.0303 (16)	0.0355 (18)	0.061 (2)	0.0114 (15)	−0.0032 (16)	−0.0035 (17)

### Ethyl 4-(4-methoxyphenyl)-2,7,7-trimethyl-5-oxo-1,4,5,6,7,8-hexahydroquinoline-3-carboxylate (II) Geometric parameters (Å, º)

**Table:**

O1A—C5A	1.235 (2)	N1B—H1B	0.87 (2)
O2A—C14A	1.210 (2)	N1B—C2B	1.387 (2)
O3A—C14A	1.343 (2)	N1B—C9B	1.363 (2)
O3A—C15A	1.449 (2)	C2B—C3B	1.350 (2)
O4A—C20A	1.367 (2)	C2B—C13B	1.506 (2)
O4A—C23A	1.424 (2)	C3B—C4B	1.518 (2)
N1A—H1A	0.82 (2)	C3B—C14B	1.474 (3)
N1A—C2A	1.386 (2)	C4B—H4B	1.0000
N1A—C9A	1.367 (2)	C4B—C10B	1.518 (2)
C2A—C3A	1.354 (2)	C4B—C17B	1.529 (2)
C2A—C13A	1.498 (2)	C5B—C6B	1.513 (3)
C3A—C4A	1.522 (2)	C5B—C10B	1.442 (2)
C3A—C14A	1.473 (2)	C6B—H6BA	0.9900
C4A—H4A	1.0000	C6B—H6BB	0.9900
C4A—C10A	1.513 (2)	C6B—C7B	1.530 (3)
C4A—C17A	1.523 (2)	C7B—C8B	1.525 (2)
C5A—C6A	1.507 (2)	C7B—C11B	1.528 (3)
C5A—C10A	1.440 (2)	C7B—C12B	1.534 (3)
C6A—H6AA	0.9900	C8B—H8BA	0.9900
C6A—H6AB	0.9900	C8B—H8BB	0.9900
C6A—C7A	1.538 (2)	C8B—C9B	1.500 (2)
C7A—C8A	1.529 (2)	C9B—C10B	1.357 (2)
C7A—C11A	1.526 (2)	C11B—H11A	0.9800
C7A—C12A	1.530 (2)	C11B—H11B	0.9800
C8A—H8AA	0.9900	C11B—H11C	0.9800
C8A—H8AB	0.9900	C12B—H12A	0.9800
C8A—C9A	1.498 (2)	C12B—H12B	0.9800
C9A—C10A	1.356 (2)	C12B—H12C	0.9800
C11A—H11D	0.9800	C13B—H13A	0.9800
C11A—H11E	0.9800	C13B—H13B	0.9800
C11A—H11F	0.9800	C13B—H13C	0.9800
C12A—H12D	0.9800	C14B—O3B	1.464 (5)
C12A—H12E	0.9800	C14B—O3C	1.305 (4)
C12A—H12F	0.9800	C17B—C18B	1.389 (3)
C13A—H13D	0.9800	C17B—C22B	1.388 (2)
C13A—H13E	0.9800	C18B—H18B	0.9500
C13A—H13F	0.9800	C18B—C19B	1.392 (3)
C15A—H15E	0.9900	C19B—H19B	0.9500
C15A—H15F	0.9900	C19B—C20B	1.386 (2)
C15A—C16A	1.499 (3)	C20B—C21B	1.387 (3)
C16A—H16G	0.9800	C21B—H21B	0.9500
C16A—H16H	0.9800	C21B—C22B	1.379 (3)
C16A—H16I	0.9800	C22B—H22B	0.9500
C17A—C18A	1.398 (2)	C23B—H23A	0.9800
C17A—C22A	1.377 (2)	C23B—H23B	0.9800
C18A—H18A	0.9500	C23B—H23C	0.9800
C18A—C19A	1.380 (2)	O3B—C15B	1.480 (5)
C19A—H19A	0.9500	C15B—H15A	0.9900
C19A—C20A	1.390 (3)	C15B—H15B	0.9900
C20A—C21A	1.385 (3)	C15B—C16B	1.495 (7)
C21A—H21A	0.9500	C16B—H16A	0.9800
C21A—C22A	1.395 (3)	C16B—H16B	0.9800
C22A—H22A	0.9500	C16B—H16C	0.9800
C23A—H23D	0.9800	O3C—C15C	1.461 (4)
C23A—H23E	0.9800	C15C—H15C	0.9900
C23A—H23F	0.9800	C15C—H15D	0.9900
O1B—C5B	1.239 (2)	C15C—C16C	1.454 (6)
O2B—C14B	1.205 (2)	C16C—H16D	0.9800
O4B—C20B	1.372 (2)	C16C—H16E	0.9800
O4B—C23B	1.424 (3)	C16C—H16F	0.9800
			
C14A—O3A—C15A	117.37 (14)	C2B—C3B—C14B	118.97 (17)
C20A—O4A—C23A	117.16 (15)	C14B—C3B—C4B	119.29 (15)
C2A—N1A—H1A	119.1 (14)	C3B—C4B—H4B	107.9
C9A—N1A—H1A	118.1 (14)	C3B—C4B—C17B	111.84 (15)
C9A—N1A—C2A	122.74 (15)	C10B—C4B—C3B	110.35 (14)
N1A—C2A—C13A	113.58 (15)	C10B—C4B—H4B	107.9
C3A—C2A—N1A	119.53 (15)	C10B—C4B—C17B	110.91 (14)
C3A—C2A—C13A	126.84 (16)	C17B—C4B—H4B	107.9
C2A—C3A—C4A	121.22 (15)	O1B—C5B—C6B	119.96 (16)
C2A—C3A—C14A	120.62 (15)	O1B—C5B—C10B	120.96 (16)
C14A—C3A—C4A	118.09 (15)	C10B—C5B—C6B	119.02 (15)
C3A—C4A—H4A	108.1	C5B—C6B—H6BA	108.5
C3A—C4A—C17A	111.21 (14)	C5B—C6B—H6BB	108.5
C10A—C4A—C3A	109.82 (13)	C5B—C6B—C7B	115.26 (15)
C10A—C4A—H4A	108.1	H6BA—C6B—H6BB	107.5
C10A—C4A—C17A	111.34 (14)	C7B—C6B—H6BA	108.5
C17A—C4A—H4A	108.1	C7B—C6B—H6BB	108.5
O1A—C5A—C6A	120.38 (15)	C6B—C7B—C12B	109.67 (16)
O1A—C5A—C10A	120.86 (16)	C8B—C7B—C6B	107.70 (14)
C10A—C5A—C6A	118.72 (14)	C8B—C7B—C11B	110.52 (15)
C5A—C6A—H6AA	108.7	C8B—C7B—C12B	108.84 (15)
C5A—C6A—H6AB	108.7	C11B—C7B—C6B	110.43 (15)
C5A—C6A—C7A	114.27 (14)	C11B—C7B—C12B	109.64 (15)
H6AA—C6A—H6AB	107.6	C7B—C8B—H8BA	109.0
C7A—C6A—H6AA	108.7	C7B—C8B—H8BB	109.0
C7A—C6A—H6AB	108.7	H8BA—C8B—H8BB	107.8
C8A—C7A—C6A	107.72 (13)	C9B—C8B—C7B	112.93 (14)
C8A—C7A—C12A	109.13 (14)	C9B—C8B—H8BA	109.0
C11A—C7A—C6A	109.63 (14)	C9B—C8B—H8BB	109.0
C11A—C7A—C8A	111.51 (14)	N1B—C9B—C8B	115.97 (15)
C11A—C7A—C12A	109.35 (14)	C10B—C9B—N1B	120.28 (16)
C12A—C7A—C6A	109.46 (14)	C10B—C9B—C8B	123.67 (16)
C7A—C8A—H8AA	108.9	C5B—C10B—C4B	119.68 (15)
C7A—C8A—H8AB	108.9	C9B—C10B—C4B	121.19 (15)
H8AA—C8A—H8AB	107.7	C9B—C10B—C5B	119.12 (16)
C9A—C8A—C7A	113.21 (14)	C7B—C11B—H11A	109.5
C9A—C8A—H8AA	108.9	C7B—C11B—H11B	109.5
C9A—C8A—H8AB	108.9	C7B—C11B—H11C	109.5
N1A—C9A—C8A	116.70 (14)	H11A—C11B—H11B	109.5
C10A—C9A—N1A	119.66 (15)	H11A—C11B—H11C	109.5
C10A—C9A—C8A	123.56 (15)	H11B—C11B—H11C	109.5
C5A—C10A—C4A	118.62 (14)	C7B—C12B—H12A	109.5
C9A—C10A—C4A	121.53 (15)	C7B—C12B—H12B	109.5
C9A—C10A—C5A	119.83 (15)	C7B—C12B—H12C	109.5
C7A—C11A—H11D	109.5	H12A—C12B—H12B	109.5
C7A—C11A—H11E	109.5	H12A—C12B—H12C	109.5
C7A—C11A—H11F	109.5	H12B—C12B—H12C	109.5
H11D—C11A—H11E	109.5	C2B—C13B—H13A	109.5
H11D—C11A—H11F	109.5	C2B—C13B—H13B	109.5
H11E—C11A—H11F	109.5	C2B—C13B—H13C	109.5
C7A—C12A—H12D	109.5	H13A—C13B—H13B	109.5
C7A—C12A—H12E	109.5	H13A—C13B—H13C	109.5
C7A—C12A—H12F	109.5	H13B—C13B—H13C	109.5
H12D—C12A—H12E	109.5	O2B—C14B—C3B	126.73 (18)
H12D—C12A—H12F	109.5	O2B—C14B—O3B	125.1 (2)
H12E—C12A—H12F	109.5	O2B—C14B—O3C	117.4 (2)
C2A—C13A—H13D	109.5	O3B—C14B—C3B	106.6 (2)
C2A—C13A—H13E	109.5	O3C—C14B—C3B	114.9 (2)
C2A—C13A—H13F	109.5	C18B—C17B—C4B	121.57 (15)
H13D—C13A—H13E	109.5	C22B—C17B—C4B	120.71 (16)
H13D—C13A—H13F	109.5	C22B—C17B—C18B	117.71 (17)
H13E—C13A—H13F	109.5	C17B—C18B—H18B	119.3
O2A—C14A—O3A	122.52 (16)	C17B—C18B—C19B	121.46 (16)
O2A—C14A—C3A	127.20 (17)	C19B—C18B—H18B	119.3
O3A—C14A—C3A	110.28 (14)	C18B—C19B—H19B	120.3
O3A—C15A—H15E	110.3	C20B—C19B—C18B	119.46 (17)
O3A—C15A—H15F	110.3	C20B—C19B—H19B	120.3
O3A—C15A—C16A	107.13 (15)	O4B—C20B—C19B	124.77 (17)
H15E—C15A—H15F	108.5	O4B—C20B—C21B	115.41 (15)
C16A—C15A—H15E	110.3	C19B—C20B—C21B	119.81 (17)
C16A—C15A—H15F	110.3	C20B—C21B—H21B	120.1
C15A—C16A—H16G	109.5	C22B—C21B—C20B	119.77 (16)
C15A—C16A—H16H	109.5	C22B—C21B—H21B	120.1
C15A—C16A—H16I	109.5	C17B—C22B—H22B	119.1
H16G—C16A—H16H	109.5	C21B—C22B—C17B	121.79 (17)
H16G—C16A—H16I	109.5	C21B—C22B—H22B	119.1
H16H—C16A—H16I	109.5	O4B—C23B—H23A	109.5
C18A—C17A—C4A	119.95 (15)	O4B—C23B—H23B	109.5
C22A—C17A—C4A	122.03 (15)	O4B—C23B—H23C	109.5
C22A—C17A—C18A	117.99 (16)	H23A—C23B—H23B	109.5
C17A—C18A—H18A	119.5	H23A—C23B—H23C	109.5
C19A—C18A—C17A	121.04 (16)	H23B—C23B—H23C	109.5
C19A—C18A—H18A	119.5	C14B—O3B—C15B	115.7 (4)
C18A—C19A—H19A	120.0	O3B—C15B—H15A	110.0
C18A—C19A—C20A	120.08 (17)	O3B—C15B—H15B	110.0
C20A—C19A—H19A	120.0	O3B—C15B—C16B	108.4 (5)
O4A—C20A—C19A	115.63 (16)	H15A—C15B—H15B	108.4
O4A—C20A—C21A	124.56 (16)	C16B—C15B—H15A	110.0
C21A—C20A—C19A	119.81 (16)	C16B—C15B—H15B	110.0
C20A—C21A—H21A	120.4	C15B—C16B—H16A	109.5
C20A—C21A—C22A	119.13 (16)	C15B—C16B—H16B	109.5
C22A—C21A—H21A	120.4	C15B—C16B—H16C	109.5
C17A—C22A—C21A	121.92 (17)	H16A—C16B—H16B	109.5
C17A—C22A—H22A	119.0	H16A—C16B—H16C	109.5
C21A—C22A—H22A	119.0	H16B—C16B—H16C	109.5
O4A—C23A—H23D	109.5	C14B—O3C—C15C	114.0 (3)
O4A—C23A—H23E	109.5	O3C—C15C—H15C	109.8
O4A—C23A—H23F	109.5	O3C—C15C—H15D	109.8
H23D—C23A—H23E	109.5	H15C—C15C—H15D	108.3
H23D—C23A—H23F	109.5	C16C—C15C—O3C	109.2 (4)
H23E—C23A—H23F	109.5	C16C—C15C—H15C	109.8
C20B—O4B—C23B	117.05 (15)	C16C—C15C—H15D	109.8
C2B—N1B—H1B	118.7 (15)	C15C—C16C—H16D	109.5
C9B—N1B—H1B	117.1 (15)	C15C—C16C—H16E	109.5
C9B—N1B—C2B	122.57 (15)	C15C—C16C—H16F	109.5
N1B—C2B—C13B	112.90 (15)	H16D—C16C—H16E	109.5
C3B—C2B—N1B	119.68 (16)	H16D—C16C—H16F	109.5
C3B—C2B—C13B	127.41 (17)	H16E—C16C—H16F	109.5
C2B—C3B—C4B	121.67 (15)		
			
O1A—C5A—C6A—C7A	153.92 (16)	O2B—C14B—O3B—C15B	−9.3 (6)
O1A—C5A—C10A—C4A	−5.0 (2)	O2B—C14B—O3C—C15C	7.8 (5)
O1A—C5A—C10A—C9A	176.67 (16)	O4B—C20B—C21B—C22B	−179.01 (16)
O4A—C20A—C21A—C22A	−178.33 (16)	N1B—C2B—C3B—C4B	5.1 (3)
N1A—C2A—C3A—C4A	−6.9 (2)	N1B—C2B—C3B—C14B	−177.98 (17)
N1A—C2A—C3A—C14A	176.06 (15)	N1B—C9B—C10B—C4B	−6.9 (3)
N1A—C9A—C10A—C4A	8.8 (2)	N1B—C9B—C10B—C5B	172.28 (16)
N1A—C9A—C10A—C5A	−172.89 (15)	C2B—N1B—C9B—C8B	166.25 (16)
C2A—N1A—C9A—C8A	−166.11 (15)	C2B—N1B—C9B—C10B	−10.6 (3)
C2A—N1A—C9A—C10A	10.6 (2)	C2B—C3B—C4B—C10B	−19.6 (2)
C2A—C3A—C4A—C10A	22.8 (2)	C2B—C3B—C4B—C17B	104.42 (19)
C2A—C3A—C4A—C17A	−100.92 (19)	C2B—C3B—C14B—O2B	−7.6 (3)
C2A—C3A—C14A—O2A	−5.7 (3)	C2B—C3B—C14B—O3B	158.7 (3)
C2A—C3A—C14A—O3A	174.96 (15)	C2B—C3B—C14B—O3C	−176.0 (3)
C3A—C4A—C10A—C5A	157.86 (15)	C3B—C4B—C10B—C5B	−158.71 (15)
C3A—C4A—C10A—C9A	−23.8 (2)	C3B—C4B—C10B—C9B	20.4 (2)
C3A—C4A—C17A—C18A	60.5 (2)	C3B—C4B—C17B—C18B	−48.7 (2)
C3A—C4A—C17A—C22A	−117.40 (18)	C3B—C4B—C17B—C22B	132.47 (17)
C4A—C3A—C14A—O2A	177.13 (18)	C3B—C14B—O3B—C15B	−175.9 (4)
C4A—C3A—C14A—O3A	−2.2 (2)	C3B—C14B—O3C—C15C	177.3 (3)
C4A—C17A—C18A—C19A	−176.55 (16)	C4B—C3B—C14B—O2B	169.4 (2)
C4A—C17A—C22A—C21A	176.66 (16)	C4B—C3B—C14B—O3B	−24.3 (3)
C5A—C6A—C7A—C8A	51.96 (19)	C4B—C3B—C14B—O3C	1.0 (4)
C5A—C6A—C7A—C11A	−69.55 (18)	C4B—C17B—C18B—C19B	−178.71 (16)
C5A—C6A—C7A—C12A	170.51 (15)	C4B—C17B—C22B—C21B	178.92 (16)
C6A—C5A—C10A—C4A	177.41 (15)	C5B—C6B—C7B—C8B	−49.4 (2)
C6A—C5A—C10A—C9A	−0.9 (2)	C5B—C6B—C7B—C11B	71.33 (19)
C6A—C7A—C8A—C9A	−48.74 (18)	C5B—C6B—C7B—C12B	−167.74 (15)
C7A—C8A—C9A—N1A	−160.18 (15)	C6B—C5B—C10B—C4B	−174.81 (15)
C7A—C8A—C9A—C10A	23.2 (2)	C6B—C5B—C10B—C9B	6.0 (2)
C8A—C9A—C10A—C4A	−174.68 (15)	C6B—C7B—C8B—C9B	50.18 (19)
C8A—C9A—C10A—C5A	3.6 (3)	C7B—C8B—C9B—N1B	157.48 (16)
C9A—N1A—C2A—C3A	−11.6 (2)	C7B—C8B—C9B—C10B	−25.8 (2)
C9A—N1A—C2A—C13A	166.11 (16)	C8B—C9B—C10B—C4B	176.56 (16)
C10A—C4A—C17A—C18A	−62.3 (2)	C8B—C9B—C10B—C5B	−4.3 (3)
C10A—C4A—C17A—C22A	119.77 (17)	C9B—N1B—C2B—C3B	11.5 (3)
C10A—C5A—C6A—C7A	−28.5 (2)	C9B—N1B—C2B—C13B	−167.51 (17)
C11A—C7A—C8A—C9A	71.59 (18)	C10B—C4B—C17B—C18B	74.9 (2)
C12A—C7A—C8A—C9A	−167.50 (14)	C10B—C4B—C17B—C22B	−103.88 (18)
C13A—C2A—C3A—C4A	175.76 (16)	C10B—C5B—C6B—C7B	22.6 (2)
C13A—C2A—C3A—C14A	−1.3 (3)	C11B—C7B—C8B—C9B	−70.53 (19)
C14A—O3A—C15A—C16A	151.27 (16)	C12B—C7B—C8B—C9B	169.02 (16)
C14A—C3A—C4A—C10A	−160.11 (14)	C13B—C2B—C3B—C4B	−176.03 (18)
C14A—C3A—C4A—C17A	76.19 (19)	C13B—C2B—C3B—C14B	0.9 (3)
C15A—O3A—C14A—O2A	4.7 (3)	C14B—C3B—C4B—C10B	163.53 (17)
C15A—O3A—C14A—C3A	−175.91 (15)	C14B—C3B—C4B—C17B	−72.5 (2)
C17A—C4A—C10A—C5A	−78.52 (19)	C14B—O3B—C15B—C16B	−91.8 (6)
C17A—C4A—C10A—C9A	99.81 (19)	C14B—O3C—C15C—C16C	169.5 (5)
C17A—C18A—C19A—C20A	−0.2 (3)	C17B—C4B—C10B—C5B	76.8 (2)
C18A—C17A—C22A—C21A	−1.3 (3)	C17B—C4B—C10B—C9B	−104.08 (19)
C18A—C19A—C20A—O4A	178.51 (16)	C17B—C18B—C19B—C20B	0.0 (3)
C18A—C19A—C20A—C21A	−1.2 (3)	C18B—C17B—C22B—C21B	0.1 (3)
C19A—C20A—C21A—C22A	1.4 (3)	C18B—C19B—C20B—O4B	179.16 (16)
C20A—C21A—C22A—C17A	−0.1 (3)	C18B—C19B—C20B—C21B	−0.4 (3)
C22A—C17A—C18A—C19A	1.5 (3)	C19B—C20B—C21B—C22B	0.6 (3)
C23A—O4A—C20A—C19A	173.05 (16)	C20B—C21B—C22B—C17B	−0.4 (3)
C23A—O4A—C20A—C21A	−7.2 (3)	C22B—C17B—C18B—C19B	0.1 (3)
O1B—C5B—C6B—C7B	−160.26 (16)	C23B—O4B—C20B—C19B	−5.9 (3)
O1B—C5B—C10B—C4B	8.1 (2)	C23B—O4B—C20B—C21B	173.67 (18)
O1B—C5B—C10B—C9B	−171.09 (17)		

### Ethyl 4-(4-methoxyphenyl)-2,7,7-trimethyl-5-oxo-1,4,5,6,7,8-hexahydroquinoline-3-carboxylate (II) Hydrogen-bond geometry (Å, º)

**Table:**

*D*—H···*A*	*D*—H	H···*A*	*D*···*A*	*D*—H···*A*
N1*A*—H1*A*···O1*B*	0.82 (2)	2.02 (2)	2.827 (2)	167 (2)
N1*B*—H1*B*···O1*A*^i^	0.87 (2)	1.95 (2)	2.8167 (19)	172 (2)

Symmetry code: (i) *x*, *y*−1, *z*.

### Ethyl 4-(3,4-dihydroxyphenyl)-2,7,7-trimethyl-5-oxo-1,4,5,6,7,8-hexahydroquinoline-3-carboxylate (III) Crystal data

**Table:**

C_21_H_25_NO_5_	*F*(000) = 792
*M**_r_* = 371.42	*D*_x_ = 1.113 Mg m^−^^3^
Monoclinic, *P*2_1_/*n*	Mo *K*α radiation, λ = 0.71073 Å
*a* = 9.2745 (3) Å	Cell parameters from 9215 reflections
*b* = 22.1655 (7) Å	θ = 2.5–28.3°
*c* = 11.3475 (4) Å	µ = 0.08 mm^−^^1^
β = 108.2014 (17)°	*T* = 100 K
*V* = 2216.03 (13) Å^3^	Plate, brown
*Z* = 4	0.64 × 0.13 × 0.06 mm

### Ethyl 4-(3,4-dihydroxyphenyl)-2,7,7-trimethyl-5-oxo-1,4,5,6,7,8-hexahydroquinoline-3-carboxylate (III) Data collection

**Table:**

Bruker SMART BREEZE CCD diffractometer	*R*_int_ = 0.046
Radiation source: 2 kW sealed X-ray tube	θ_max_ = 28.4°, θ_min_ = 2.5°
φ and ω scans	*h* = −12→12
40175 measured reflections	*k* = −29→29
5517 independent reflections	*l* = −15→15
4263 reflections with *I* > 2σ(*I*)	

### Ethyl 4-(3,4-dihydroxyphenyl)-2,7,7-trimethyl-5-oxo-1,4,5,6,7,8-hexahydroquinoline-3-carboxylate (III) Refinement

**Table:**

Refinement on *F*^2^	Primary atom site location: structure-invariant direct methods
Least-squares matrix: full	Hydrogen site location: mixed
*R*[*F*^2^ > 2σ(*F*^2^)] = 0.045	H atoms treated by a mixture of independent and constrained refinement
*wR*(*F*^2^) = 0.123	*w* = 1/[σ^2^(*F*_o_^2^) + (0.0628*P*)^2^ + 0.7593*P*] where *P* = (*F*_o_^2^ + 2*F*_c_^2^)/3
*S* = 1.04	(Δ/σ)_max_ < 0.001
5517 reflections	Δρ_max_ = 0.41 e Å^−^^3^
260 parameters	Δρ_min_ = −0.21 e Å^−^^3^
0 restraints	

### Ethyl 4-(3,4-dihydroxyphenyl)-2,7,7-trimethyl-5-oxo-1,4,5,6,7,8-hexahydroquinoline-3-carboxylate (III) Special details

**Table:**

Geometry. All esds (except the esd in the dihedral angle between two l.s. planes) are estimated using the full covariance matrix. The cell esds are taken into account individually in the estimation of esds in distances, angles and torsion angles; correlations between esds in cell parameters are only used when they are defined by crystal symmetry. An approximate (isotropic) treatment of cell esds is used for estimating esds involving l.s. planes.
Refinement. Disordered hexanes molecules were identified in the final stage of refinement. The disorder of the hexanes was dealt with by application of the Olex2/smtbx_masks (Rees, *et al.*, 2005), which allows for the mathematical compensation of the electron contribution of disordered solvent contained in the voids to the calculated diffraction intensities.

### Ethyl 4-(3,4-dihydroxyphenyl)-2,7,7-trimethyl-5-oxo-1,4,5,6,7,8-hexahydroquinoline-3-carboxylate (III) Fractional atomic coordinates and isotropic or equivalent isotropic displacement parameters (Å^2^)

**Table:**

	*x*	*y*	*z*	*U*_iso_*/*U*_eq_	
O1	1.16874 (11)	0.16574 (4)	0.51737 (9)	0.0186 (2)	
O2	0.97871 (12)	0.34503 (5)	0.68766 (9)	0.0208 (2)	
O4	0.45936 (11)	0.14086 (5)	0.41764 (10)	0.0240 (2)	
O5	0.49133 (12)	0.12980 (5)	0.65976 (10)	0.0218 (2)	
O3	0.87314 (12)	0.41878 (4)	0.55338 (9)	0.0216 (2)	
N1	0.78891 (13)	0.29217 (5)	0.26502 (10)	0.0156 (2)	
C5	1.08644 (15)	0.18180 (6)	0.41329 (12)	0.0149 (3)	
C8	0.89609 (15)	0.21511 (6)	0.16440 (12)	0.0160 (3)	
H8A	0.958420	0.240536	0.127373	0.019*	
H8B	0.792707	0.212750	0.104128	0.019*	
C4	0.93969 (14)	0.25570 (6)	0.50871 (11)	0.0139 (3)	
H4A	1.037134	0.257551	0.578628	0.017*	
C6	1.10852 (15)	0.15446 (6)	0.29827 (12)	0.0174 (3)	
H6A	1.148756	0.113046	0.318085	0.021*	
H6B	1.185931	0.178302	0.275254	0.021*	
C17	0.82560 (15)	0.21896 (6)	0.55115 (12)	0.0149 (3)	
C18	0.69686 (15)	0.19536 (6)	0.46308 (12)	0.0160 (3)	
H18	0.684531	0.200209	0.377301	0.019*	
C22	0.84222 (16)	0.21058 (6)	0.67665 (12)	0.0177 (3)	
H22	0.930164	0.225397	0.737945	0.021*	
C20	0.60235 (15)	0.15836 (6)	0.62458 (13)	0.0173 (3)	
C9	0.88732 (14)	0.24475 (6)	0.28105 (12)	0.0145 (3)	
C2	0.79999 (15)	0.33426 (6)	0.35723 (12)	0.0155 (3)	
C19	0.58740 (15)	0.16513 (6)	0.49897 (12)	0.0169 (3)	
C3	0.88223 (15)	0.31999 (6)	0.47602 (12)	0.0149 (3)	
C13	0.71604 (17)	0.39170 (7)	0.30927 (13)	0.0215 (3)	
H13A	0.788056	0.422485	0.300887	0.032*	
H13B	0.664522	0.405840	0.367496	0.032*	
H13C	0.640811	0.384230	0.228143	0.032*	
C10	0.97144 (14)	0.22778 (6)	0.39745 (12)	0.0145 (3)	
C14	0.91478 (15)	0.36129 (6)	0.58100 (12)	0.0158 (3)	
C12	1.00654 (17)	0.13217 (7)	0.07145 (13)	0.0222 (3)	
H12A	0.914860	0.130802	−0.000786	0.033*	
H12B	1.053357	0.092077	0.085603	0.033*	
H12C	1.078437	0.161201	0.056243	0.033*	
C7	0.96430 (15)	0.15158 (6)	0.18598 (12)	0.0163 (3)	
C21	0.73077 (16)	0.18065 (7)	0.71252 (12)	0.0185 (3)	
H21	0.742940	0.175475	0.798193	0.022*	
C11	0.85119 (17)	0.10672 (7)	0.20946 (14)	0.0224 (3)	
H11A	0.825571	0.118927	0.283507	0.034*	
H11B	0.896573	0.066356	0.222225	0.034*	
H11C	0.758898	0.106099	0.137716	0.034*	
C15	0.90744 (19)	0.46064 (7)	0.65759 (14)	0.0261 (3)	
H15A	1.018040	0.461676	0.701111	0.031*	
H15B	0.854928	0.448188	0.717373	0.031*	
C16	0.8524 (2)	0.52160 (7)	0.60416 (16)	0.0379 (4)	
H16A	0.899073	0.531841	0.540303	0.057*	
H16B	0.880369	0.551964	0.670189	0.057*	
H16C	0.741720	0.520718	0.567149	0.057*	
H1	0.741 (2)	0.3036 (8)	0.1889 (17)	0.024 (4)*	
H4	0.467 (2)	0.1431 (10)	0.343 (2)	0.043 (6)*	
H5	0.412 (3)	0.1256 (10)	0.597 (2)	0.050 (6)*	

### Ethyl 4-(3,4-dihydroxyphenyl)-2,7,7-trimethyl-5-oxo-1,4,5,6,7,8-hexahydroquinoline-3-carboxylate (III) Atomic displacement parameters (Å^2^)

**Table:**

	*U*^11^	*U*^22^	*U*^33^	*U*^12^	*U*^13^	*U*^23^
O1	0.0193 (5)	0.0216 (5)	0.0118 (5)	0.0028 (4)	0.0004 (4)	0.0001 (4)
O2	0.0256 (5)	0.0223 (5)	0.0116 (5)	0.0036 (4)	0.0016 (4)	−0.0014 (4)
O4	0.0182 (5)	0.0382 (6)	0.0143 (5)	−0.0089 (4)	0.0033 (4)	−0.0007 (5)
O5	0.0209 (5)	0.0285 (6)	0.0175 (5)	−0.0020 (4)	0.0084 (4)	0.0032 (4)
O3	0.0317 (6)	0.0170 (5)	0.0131 (5)	0.0018 (4)	0.0024 (4)	−0.0023 (4)
N1	0.0169 (5)	0.0181 (6)	0.0089 (5)	0.0018 (4)	0.0000 (4)	0.0000 (4)
C5	0.0150 (6)	0.0160 (6)	0.0128 (6)	−0.0027 (5)	0.0031 (5)	−0.0007 (5)
C8	0.0165 (6)	0.0198 (7)	0.0101 (6)	0.0003 (5)	0.0018 (5)	−0.0002 (5)
C4	0.0132 (6)	0.0173 (6)	0.0090 (6)	0.0014 (5)	0.0002 (5)	−0.0004 (5)
C6	0.0154 (6)	0.0216 (7)	0.0135 (6)	0.0037 (5)	0.0020 (5)	−0.0017 (5)
C17	0.0159 (6)	0.0148 (6)	0.0135 (6)	0.0027 (5)	0.0041 (5)	0.0010 (5)
C18	0.0167 (6)	0.0199 (7)	0.0109 (6)	0.0018 (5)	0.0035 (5)	0.0014 (5)
C22	0.0197 (6)	0.0192 (7)	0.0123 (6)	0.0011 (5)	0.0021 (5)	−0.0009 (5)
C20	0.0187 (6)	0.0179 (7)	0.0163 (7)	0.0025 (5)	0.0068 (5)	0.0024 (5)
C9	0.0134 (6)	0.0161 (6)	0.0136 (6)	−0.0030 (5)	0.0034 (5)	−0.0010 (5)
C2	0.0152 (6)	0.0170 (7)	0.0141 (6)	−0.0008 (5)	0.0042 (5)	0.0003 (5)
C19	0.0154 (6)	0.0192 (7)	0.0142 (6)	0.0016 (5)	0.0021 (5)	0.0002 (5)
C3	0.0148 (6)	0.0168 (7)	0.0123 (6)	0.0000 (5)	0.0033 (5)	−0.0003 (5)
C13	0.0264 (7)	0.0210 (7)	0.0148 (7)	0.0053 (6)	0.0031 (6)	0.0016 (5)
C10	0.0148 (6)	0.0162 (6)	0.0115 (6)	−0.0013 (5)	0.0030 (5)	−0.0008 (5)
C14	0.0143 (6)	0.0178 (7)	0.0147 (6)	−0.0005 (5)	0.0037 (5)	−0.0002 (5)
C12	0.0233 (7)	0.0277 (8)	0.0147 (6)	0.0043 (6)	0.0048 (6)	−0.0035 (6)
C7	0.0161 (6)	0.0197 (7)	0.0120 (6)	0.0011 (5)	0.0027 (5)	−0.0018 (5)
C21	0.0231 (7)	0.0210 (7)	0.0113 (6)	0.0034 (5)	0.0050 (5)	0.0018 (5)
C11	0.0239 (7)	0.0213 (7)	0.0210 (7)	−0.0028 (6)	0.0054 (6)	−0.0038 (6)
C15	0.0369 (9)	0.0198 (7)	0.0168 (7)	0.0017 (6)	0.0016 (6)	−0.0048 (6)
C16	0.0608 (12)	0.0201 (8)	0.0272 (8)	0.0067 (8)	0.0055 (8)	−0.0037 (7)

### Ethyl 4-(3,4-dihydroxyphenyl)-2,7,7-trimethyl-5-oxo-1,4,5,6,7,8-hexahydroquinoline-3-carboxylate (III) Geometric parameters (Å, º)

**Table:**

O1—C5	1.2413 (16)	C18—C19	1.3798 (19)
O2—C14	1.2240 (16)	C22—H22	0.9500
O4—C19	1.3662 (16)	C22—C21	1.392 (2)
O4—H4	0.87 (2)	C20—C19	1.3964 (19)
O5—C20	1.3701 (17)	C20—C21	1.3842 (19)
O5—H5	0.85 (2)	C9—C10	1.3605 (18)
O3—C14	1.3400 (17)	C2—C3	1.3625 (18)
O3—C15	1.4578 (17)	C2—C13	1.5029 (19)
N1—C9	1.3666 (17)	C3—C14	1.4573 (18)
N1—C2	1.3814 (17)	C13—H13A	0.9800
N1—H1	0.876 (18)	C13—H13B	0.9800
C5—C6	1.5099 (18)	C13—H13C	0.9800
C5—C10	1.4453 (19)	C12—H12A	0.9800
C8—H8A	0.9900	C12—H12B	0.9800
C8—H8B	0.9900	C12—H12C	0.9800
C8—C9	1.5026 (18)	C12—C7	1.5316 (19)
C8—C7	1.5317 (19)	C7—C11	1.528 (2)
C4—H4A	1.0000	C21—H21	0.9500
C4—C17	1.5275 (18)	C11—H11A	0.9800
C4—C3	1.5260 (19)	C11—H11B	0.9800
C4—C10	1.5153 (18)	C11—H11C	0.9800
C6—H6A	0.9900	C15—H15A	0.9900
C6—H6B	0.9900	C15—H15B	0.9900
C6—C7	1.5336 (18)	C15—C16	1.504 (2)
C17—C18	1.3973 (18)	C16—H16A	0.9800
C17—C22	1.3961 (18)	C16—H16B	0.9800
C18—H18	0.9500	C16—H16C	0.9800
			
C19—O4—H4	108.5 (14)	C2—C3—C4	120.15 (12)
C20—O5—H5	109.9 (15)	C2—C3—C14	124.88 (12)
C14—O3—C15	116.21 (11)	C14—C3—C4	114.86 (11)
C9—N1—C2	122.01 (11)	C2—C13—H13A	109.5
C9—N1—H1	117.7 (12)	C2—C13—H13B	109.5
C2—N1—H1	116.6 (12)	C2—C13—H13C	109.5
O1—C5—C6	119.89 (12)	H13A—C13—H13B	109.5
O1—C5—C10	122.08 (12)	H13A—C13—H13C	109.5
C10—C5—C6	118.01 (11)	H13B—C13—H13C	109.5
H8A—C8—H8B	107.7	C5—C10—C4	120.82 (11)
C9—C8—H8A	108.9	C9—C10—C5	119.53 (12)
C9—C8—H8B	108.9	C9—C10—C4	119.62 (12)
C9—C8—C7	113.23 (11)	O2—C14—O3	121.77 (12)
C7—C8—H8A	108.9	O2—C14—C3	122.42 (13)
C7—C8—H8B	108.9	O3—C14—C3	115.80 (11)
C17—C4—H4A	108.4	H12A—C12—H12B	109.5
C3—C4—H4A	108.4	H12A—C12—H12C	109.5
C3—C4—C17	110.54 (11)	H12B—C12—H12C	109.5
C10—C4—H4A	108.4	C7—C12—H12A	109.5
C10—C4—C17	112.27 (11)	C7—C12—H12B	109.5
C10—C4—C3	108.83 (11)	C7—C12—H12C	109.5
C5—C6—H6A	108.6	C8—C7—C6	107.70 (11)
C5—C6—H6B	108.6	C12—C7—C8	108.96 (11)
C5—C6—C7	114.61 (11)	C12—C7—C6	109.20 (11)
H6A—C6—H6B	107.6	C11—C7—C8	110.75 (11)
C7—C6—H6A	108.6	C11—C7—C6	110.50 (11)
C7—C6—H6B	108.6	C11—C7—C12	109.67 (12)
C18—C17—C4	119.69 (11)	C22—C21—H21	119.7
C22—C17—C4	121.73 (12)	C20—C21—C22	120.64 (13)
C22—C17—C18	118.51 (12)	C20—C21—H21	119.7
C17—C18—H18	119.5	C7—C11—H11A	109.5
C19—C18—C17	120.91 (12)	C7—C11—H11B	109.5
C19—C18—H18	119.5	C7—C11—H11C	109.5
C17—C22—H22	119.8	H11A—C11—H11B	109.5
C21—C22—C17	120.41 (12)	H11A—C11—H11C	109.5
C21—C22—H22	119.8	H11B—C11—H11C	109.5
O5—C20—C19	120.20 (12)	O3—C15—H15A	110.4
O5—C20—C21	120.70 (12)	O3—C15—H15B	110.4
C21—C20—C19	119.10 (13)	O3—C15—C16	106.51 (12)
N1—C9—C8	115.90 (11)	H15A—C15—H15B	108.6
C10—C9—N1	119.90 (12)	C16—C15—H15A	110.4
C10—C9—C8	124.20 (12)	C16—C15—H15B	110.4
N1—C2—C13	112.97 (11)	C15—C16—H16A	109.5
C3—C2—N1	118.61 (12)	C15—C16—H16B	109.5
C3—C2—C13	128.42 (13)	C15—C16—H16C	109.5
O4—C19—C18	123.80 (12)	H16A—C16—H16B	109.5
O4—C19—C20	115.81 (12)	H16A—C16—H16C	109.5
C18—C19—C20	120.38 (12)	H16B—C16—H16C	109.5
			
O1—C5—C6—C7	150.87 (13)	C22—C17—C18—C19	0.8 (2)
O1—C5—C10—C4	−4.0 (2)	C9—N1—C2—C3	−17.94 (19)
O1—C5—C10—C9	178.22 (13)	C9—N1—C2—C13	161.87 (12)
O5—C20—C19—O4	−1.13 (19)	C9—C8—C7—C6	−47.01 (15)
O5—C20—C19—C18	178.15 (13)	C9—C8—C7—C12	−165.35 (11)
O5—C20—C21—C22	−178.88 (13)	C9—C8—C7—C11	73.93 (14)
N1—C9—C10—C5	−173.59 (12)	C2—N1—C9—C8	−160.56 (12)
N1—C9—C10—C4	8.57 (19)	C2—N1—C9—C10	18.4 (2)
N1—C2—C3—C4	−9.26 (19)	C2—C3—C14—O2	173.96 (13)
N1—C2—C3—C14	174.56 (12)	C2—C3—C14—O3	−7.4 (2)
C5—C6—C7—C8	53.26 (15)	C19—C20—C21—C22	1.4 (2)
C5—C6—C7—C12	171.44 (12)	C3—C4—C17—C18	79.11 (15)
C5—C6—C7—C11	−67.84 (15)	C3—C4—C17—C22	−97.69 (14)
C8—C9—C10—C5	5.3 (2)	C3—C4—C10—C5	151.08 (12)
C8—C9—C10—C4	−172.52 (12)	C3—C4—C10—C9	−31.10 (16)
C4—C17—C18—C19	−176.06 (12)	C13—C2—C3—C4	170.96 (13)
C4—C17—C22—C21	175.27 (12)	C13—C2—C3—C14	−5.2 (2)
C4—C3—C14—O2	−2.40 (19)	C10—C5—C6—C7	−31.05 (17)
C4—C3—C14—O3	176.26 (11)	C10—C4—C17—C18	−42.63 (16)
C6—C5—C10—C4	178.00 (12)	C10—C4—C17—C22	140.57 (13)
C6—C5—C10—C9	0.18 (19)	C10—C4—C3—C2	31.57 (16)
C17—C4—C3—C2	−92.17 (14)	C10—C4—C3—C14	−151.88 (11)
C17—C4—C3—C14	84.38 (13)	C14—O3—C15—C16	179.42 (14)
C17—C4—C10—C5	−86.21 (14)	C7—C8—C9—N1	−160.98 (11)
C17—C4—C10—C9	91.60 (15)	C7—C8—C9—C10	20.07 (19)
C17—C18—C19—O4	−179.79 (13)	C21—C20—C19—O4	178.62 (13)
C17—C18—C19—C20	1.0 (2)	C21—C20—C19—C18	−2.1 (2)
C17—C22—C21—C20	0.5 (2)	C15—O3—C14—O2	−0.38 (19)
C18—C17—C22—C21	−1.6 (2)	C15—O3—C14—C3	−179.05 (12)

### Ethyl 4-(3,4-dihydroxyphenyl)-2,7,7-trimethyl-5-oxo-1,4,5,6,7,8-hexahydroquinoline-3-carboxylate (III) Hydrogen-bond geometry (Å, º)

**Table:**

*D*—H···*A*	*D*—H	H···*A*	*D*···*A*	*D*—H···*A*
N1—H1···O1^i^	0.876 (18)	1.971 (19)	2.8378 (15)	169.8 (16)
O4—H4···O2^i^	0.87 (2)	1.82 (2)	2.6894 (14)	175 (2)
O5—H5···O1^ii^	0.85 (2)	2.33 (2)	3.0293 (14)	140 (2)

Symmetry codes: (i) *x*−1/2, −*y*+1/2, *z*−1/2; (ii) *x*−1, *y*, *z*.
